# Phytoremediation of Wastewater From Oil Well Drilling: Potential of Different Plant Species

**DOI:** 10.1002/wer.70385

**Published:** 2026-04-26

**Authors:** Thiago Oliveira de Souza, Tiago Antônio de Oliveira Mendes, Larissa Cassemiro Pacheco Monteiro, Luciano Endrigo Watthier Júnior, Diego da Silva Marques, Alisson Carraro Borges

**Affiliations:** ^1^ Department of Agricultural Engineering Federal University of Viçosa Viçosa Brazil; ^2^ Department of Biochemistry and Molecular Biology Federal University of Viçosa Viçosa Brazil

**Keywords:** constructed wetlands, drilling waste, enzymatic activity, hydrocarbons, phytoremediation

## Abstract

This study evaluated the removal of contaminants present in wastewater from oil well drilling in the presence of three plant species: water hyacinth ( *Eichhornia crassipes* ), cattail ( *Typha domingensis* ), and purple fountain grass ( *Cenchrus setaceus* “Rubrum”). The synthesized wastewater contained potentially toxic metals (Zn, Cu, and Cr) and representative hydrocarbons (naphthalene, hexane, and hexadecane) and it was applied in hydroponic systems operating in sequential batches. Agronomic, biochemical, and water quality variables were monitored periodically. The results indicated that *C. setaceus* had the best overall performance, indicating its absence of mortality, agronomic stability, and statistically significant removal of color, biochemical oxygen demand, total organic carbon, total carbon, and metals. The kinetics of chemical oxygen demand removal were faster in the presence of *C. setaceus* , with a higher reaction coefficient and better fit of the first‐order model with residual (plateau) to the data. Although they promoted the removal of some pollutants, the performance of the treatments in the presence of *T. domingensis* and *E. crassipes* was inferior to that of the treatment with *C. setaceus* in terms of stability, vigor, and consistency of the results. It was concluded that *C. setaceus* has high tolerance to contaminated environments and potential for application in constructed wetland systems for the treatment of wastewater from well drilling and is a promising alternative for sustainable phytoremediation strategies.

## Summary

-
*Cenchrus setaceus* showed highest stability and contaminant removal in hydroponic treatment of oil well drilling wastewater.
- Purple fountain grass effectively removed color, BOD, TOC, total carbon, and metals without plant mortality.
- Faster COD removal kinetics indicate strong potential for efficient organic load reduction in engineered treatment systems.
- Water hyacinth and cattail were less stable, limiting their reliability for high‐strength industrial wastewaters.
-
*C. setaceus* is a promising macrophyte for constructed wetlands treating complex oil‐drilling effluents.

## Graphical abstract

Purple fountain grass ( *Cenchrus setaceus* “Rubrum”) is a promising plant for using in constructed wetlands treating complex oil drilling effluents.

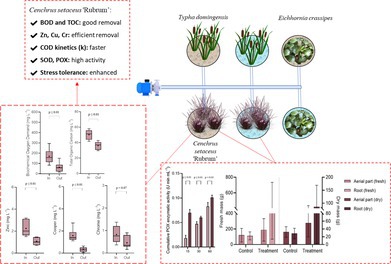

## 1 Introduction

The oil exploration and production industry is one of the activities that generates the most highly complex waste with a potential environmental impact (Ahsan 2024). Among these wastes, drilling fluids stand out and are used in virtually all the phases of well drilling. These fluids perform important functions such as stabilizing the well walls, controlling hydrostatic pressure, cooling and lubricating the drill bit, and transporting the cuttings generated during drilling (Deville 2022).

From an environmental point of view, liquid waste from the use of these fluids has a complex and heterogeneous composition, presenting a combination of water (or oil), fine solids (such as bentonite and gravel), barite, and residues from the chemical additives used (Marinho et al. 2024). Its composition may include metals, such as chromium (Cr), zinc (Zn), copper (Cu), and lead (Pb), which may be associated with barite, suspended solids, and grease. There is also a possibility of contamination by petroleum‐derived hydrocarbons (Agnihotri et al. 2023).

According to data from the Brazilian oil industry, the volumes generated were significant. In 2024, 1800 t of gravel and water‐based fluids and 800 t of nonaqueous fluid waste were reported to have been generated (Petrobras 2025). These figures indicate that a huge amount of drilling fluid are handled annually, highlighting the importance of proper management.

Nature‐based solutions, such as constructed wetland (CW) systems, have been widely used for wastewater treatment because of their sustainability, low operating costs, and potential for resource recovery (Gonzalez‐Flo et al. 2023; Pandiarajan and Sankararajan 2025). The efficiency of these systems depends on several factors, particularly the choice of plant species and packing material. Pollutant removal in CWs is strongly related to the phytoremediation capacity of plants, which acts through different mechanisms (Pandey et al. 2021).

Phytoremediation encompasses processes such as phytodegradation, in which plant enzymes promote the decomposition of organic contaminants into less toxic or inorganic compounds such as carbon dioxide and water (Ismail and Talebi 2023). Conversion of these pollutants into stable intermediates, which are stored in plant tissues, can also occur (Thakur et al. 2023). Another important mechanism is bioremediation, which is carried out by microorganisms present in the biofilm formed in the filling material and is also associated with the root zone, contributing to the degradation of toxic substances (Pandey et al. 2021). Phytoextraction, in turn, involves the absorption of metals or other inorganic compounds by plants, with their accumulation and translocation in tissues, allowing subsequent removal by harvesting and treatment of biomass (Thakur et al. 2023).

In phytoremediation, the antioxidant system of plants plays a key role in protecting against the toxic effects of oxidative stress, usually triggered by the accumulation of reactive oxygen species (ROS), such as superoxide radicals (O_2_^−^) and hydrogen peroxide (H_2_O_2_), which can damage proteins, lipids, DNA, and other cellular structures, compromising plant growth and vitality (Borges et al. 2023). To neutralize these molecules, plants activate a coordinated set of antioxidant enzymes, among which superoxide dismutase (SOD) is responsible for converting superoxide radicals into H_2_O_2_ (Mushtaq et al. 2022); catalase (CAT), which decomposes hydrogen peroxide into water and molecular oxygen (Batool et al. 2022); and peroxidases (POX), which participate in the decomposition of peroxides, promote the oxidation of phenolic compounds, and are involved in structural processes such as lignification and suberization, contributing to the strengthening of cell walls (Khavari‐Nejad 2019; Wang et al. 2018). The activation of these enzymes is a common adaptive response in plants exposed to contaminated environments, with a coordinated increase in SOD, CAT, and POX activities, indicating mobilization of the antioxidant defense system in response to pollutant‐induced stress.

In view of the above, the present study aimed to select the plant species with the best performance in the treatment and highest tolerance to stress conditions caused by this type of waste. To this end, three plant species were evaluated after they were subjected to synthetic wastewater‐containing metals (Zn, Cu, and Cr) and hydrocarbons representative of different classes: hexadecane (long chain), hexane (short chain), and naphthalene (aromatic). The comparison between species was based on agronomic and biochemical indicators and on the efficiency of pollutant removal, allowing the identification of the most promising plant for application in nature‐based solutions treating wastewater from oil well drilling.

## 2 Materials and Methods

### 2.1 Location and Preparation of the Effluent to Be Treated

To simulate the characteristics of liquid waste from oil well drilling, a synthetic solution was prepared in the laboratory and then applied to phytoremediation systems at a rate of 40 L per experimental unit per batch.

The organic fraction consisted of three hydrocarbons representative of the different classes of contaminants present in petroleum: hexadecane (C_16_H_34_), a long‐chain alkane; hexane (C_6_H_14_), a short‐chain alkane; and naphthalene, a two‐ring polycyclic aromatic hydrocarbon. Hexadecane was included based on studies on CW applied to the bioremediation of total petroleum hydrocarbons (TPH) (Watzinger et al. 2021), in addition to being recommended as a standard in USEPA tests, such as in method 1664 (20 mg L^−1^). The concentration used was approximately 30 mg; the wastewater was applied in a volume of 40 L per experimental unit per batch. Hexane, although less cited in the literature, was used at approximately 60 mg L^−1^ to represent light fractions. Naphthalene was included at approximately 50 mg L^−1^ to represent the aromatic compounds typical of oil‐contaminated water (Amakiri et al. 2022; Watzinger et al. 2021). Naphthalene was chosen over other aromatic compounds such as benzene, toluene, ethylbenzene, and xylene (BTEX) because of its lower occupational toxicity, which ensures greater safety for researchers during the handling and application of the synthetic effluent.

Regarding the inorganic fraction, zinc sulfate (ZnSO_4_), copper sulfate (CuSO_4_), and chromium chloride (CrCl_3_) were added to compose metals commonly found in drilling waste and recognized for their toxic potential. Concentrations varied during the experiment due to dilution, adsorption, and system dynamics in the field, with values ranging from 2 to 6 mg L^−1^ for Zn, 1.5 to 5 mg L^−1^ for Cu, and 0.5 to 1.5 mg L^−1^ for Cr. These values were defined based on previous studies that used contaminated synthetic water, such as the work by Wagner et al. (2021), which applied ZnCl_2_ to obtain 8 mg L^−1^ of Zn and aimed to simulate critical environmental contamination scenarios. Simultaneously, kaolin was added to the influent to ensure greater representativeness of the suspended solids and turbidity.

The pH of the synthesized wastewater was measured prior to its application in each batch. The influent presented a slightly acidic to near‐neutral condition, with a mean pH of 6.82 ± 0.20 (*n* = 21). This pH range is representative of oil well drilling wastewater and is relevant for controlling metal speciation, microbial activity, and phytoremediation processes.

### 2.2 Phytoremediation

The experiment was conducted with the cultivation of three plant species: purple fountain grass ( *Cenchrus setaceus* “Rubrum”), cattail ( *Typha domingensis* ), and water hyacinth ( *Eichhornia crassipes* ). Seedlings were individually placed in HDPE reservoirs with a useful volume of 40 L each and adapted to operate under hydroponic conditions.

The species were selected based on complementary phytoremediation and ecological characteristics. *E. crassipes* was chosen due to its well‐documented pollutant removal capacity in aquatic environments. *T. domingensis* was selected because of its widespread application in constructed wetlands and tolerance to variable environmental conditions. *C. setaceus* was included due to its salinity tolerance and its potential applicability in future treatment stages involving higher saline loads, as well as its ornamental suitability in landscape‐integrated systems.

*E. crassipes* and *T. domingensis* individuals were collected from natural environments, whereas *C. setaceus* plants were obtained from a commercial nursery. All plants consisted of mature individuals (not first‐ or second‐generation experimental offspring) and were acclimated in a greenhouse with water and nutrient supplementation prior to wastewater exposure to ensure physiological stabilization and experimental uniformity.

For *C. setaceus* and *T. domingensis* , a support system was developed using Styrofoam plates, allowing the plants to be supported with immersed roots. *E. crassipes* , a floating macrophyte, does not require additional support. Each reservoir was equipped with a tap installed at its base to enable drainage and sample collection.

To maintain the supply of oxygen to the roots, a forced aeration system was installed and operated for 1 h per day. This system consisted of an air compressor connected to a main pipe from which branches with hoses were directed to the bottom of each derived unit. Porous stones were attached to each end, allowing the formation of microbubbles and promoting the oxygenation of the liquid medium.

During the initial acclimatization phase of the plants, Hoagland nutrient solution at 25% of the original concentration was used. After the adaptation period, the experiment began with the distribution of treatments, with two reservoirs (57 × 32 × 32 cm) allocated per plant species: one for the control group, containing only Hoagland solution (at this point at 15% strength), and another for the treatment group, which received the contaminated synthetic solution, in addition to the same nutrient solution diluted weekly.

**FIGURE 1:**
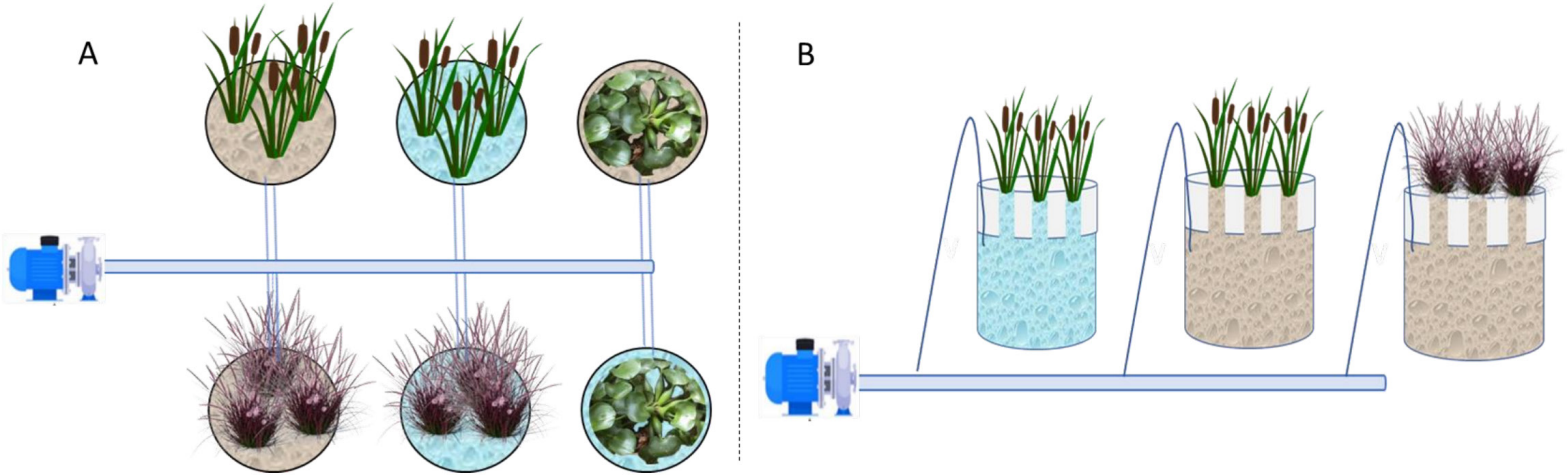
Schematic diagram of the experimental phytoremediation systems used in this study. (A) Top view and (B) side view of the 40‐L hydroponic reservoirs containing three individuals of each plant species ( *Eichhornia crassipes* , *Typha domingensis* , or *Cenchrus setaceus* “Rubrum”). Each species was cultivated under two conditions: control (15% Hoagland nutrient solution) and treatment (synthetic wastewater from oil well drilling + 15% Hoagland solution). The system included forced aeration and bottom drainage for sampling.

Each reservoir received three individuals of the same species, totaling six plants per species (three in the control and three in the wastewater treatments). Figure 1 shows the schematic of the experimental setup.

### 2.3 Monitoring Variables

The experiment was conducted over 70 days, during which the plants were exposed to the synthetic effluent and the variables evaluated were monitored. The duration of each batch, equivalent to the hydraulic retention time (HRT), was 7 days, that is, the synthetic effluent was added and remained in contact with the plants during this period. During each cycle (batch), samples were collected daily for routine variable monitoring, and at the end of the 7 days, the final sampling was performed for weekly analysis.

Owing to small variations in the concentrations of the synthetic wastewater prepared in the laboratory, each batch of this raw effluent applied to a given plant species was analyzed separately, allowing for a correct comparison between the applied and treated liquids. Analyses of the raw and treated samples were performed according to the procedures described in the Standard Methods for the Examination of Water and Wastewater (APHA 2023).

The variables monitored daily included redox potential (Eh), pH, turbidity, color, electrical conductivity, and temperature using a portable multiparameter device.

Weekly analyses included biochemical oxygen demand (BOD), total organic carbon (TOC), total carbon (TC), metal concentrations (Zn, Cu, and Cr), total solids (TS), and total suspended solids (TSS). Chemical oxygen demand (COD) was determined at an intermediate frequency, three times a week, to better monitor the degradation of organic matter. Both BOD and COD analyses were performed on unfiltered (raw) samples.

During the experiment, the agronomic variables of the plants, height of the aerial part, and root length were monitored weekly, measured with the aid of a tape measure. At the end of the experimental period, all plants were harvested and sectioned into aerial parts and root systems for subsequent determination of biomass.

The fresh mass of each fraction was recorded using a precision scale, and the samples were placed in an air‐circulating oven and maintained at 65°C for 72 h. This process allowed for gradual evaporation of water from the samples. At the end of the drying period, the samples were weighed again to determine the dry mass of aerial parts and roots.

Because the experiment was conducted at pilot scale (40‐L hydroponic reactors) under greenhouse conditions, influent samples were collected individually from each reactor at the beginning of every batch cycle. Slight variations in measured influent pollutant concentrations may have occurred due to residual adsorption from previous batches to plant tissues or reactor surfaces, followed by partial desorption at the start of subsequent cycles. Therefore, concentration factors were calculated using the specific influent measured in each reactor rather than assuming a uniform influent concentration across treatments. Statistical analysis (Tukey test) confirmed that influent concentrations did not differ significantly among treatments.

### 2.4 Enzymatic Activity (SOD, CAT, and POX)

#### 2.4.1 Preparation of the Enzyme Extract

The plant tissue samples were macerated in liquid nitrogen and homogenized in 2 mL of 0.1 mol L^−1^ phosphate buffer (pH 6.8) containing 1 mmol L^−1^ of phenylmethylsulfonyl fluoride, 0.1 mmol L^−1^ EDTA, and 0.02 g polyvinylpolypyrrolidone to inhibit proteases and eliminate phenolic compounds that could interfere with the analyses. Throughout the process, samples were stored in nitrogen to preserve protein integrity.

After homogenization, the extracts were centrifuged at 15,000 rpm for 15 min at 4°C. The obtained supernatant (enzymatic extract) was stored in a refrigerator and used immediately for the analysis of enzymatic activities.

#### 2.4.2 SOD Activity

SOD activity was determined based on its ability to inhibit the reduction of nitroblue tetrazolium (NBT) by the generation of superoxide radicals. The reaction mixture contained 10 μL of sodium phosphate buffer (50 mmol L^−1^, pH 7.0), 33.3 μL of EDTA (0.1 mmol L^−1^), 20 μL of riboflavin (10 μmol L^−1^), 48 μL of methionine (12 mmol L^−1^), 20 μL of NBT (50 μmol L^−1^), 20 μL of carbonate (50 mmol L^−1^), and 6.6 μL of the enzyme extract. The reaction was exposed to fluorescent light for 15 min, and formazan formation was measured by spectrophotometry at 560 nm. One unit of SOD was defined as the amount of enzyme required to cause a 50% inhibition of NBT reduction.

#### 2.4.3 Peroxidase Activity

POX activity was evaluated using guaiacol as a substrate and tetraguaiacol formation was monitored by spectrophotometry at 470 nm. The reaction mixture consisted of 180 μL of phosphate buffer (50 mmol L^−1^, pH 6.8), 10 μL of guaiacol (20 mmol L^−1^), 10 μL of H_2_O_2_ (30 mmol L^−1^), and 10 μL of the enzyme extract. Readings were taken every 15 s for 5 min. Activity was expressed as the change in absorbance per minute per milliliter of the extract.

#### 2.4.4 CAT Activity

CAT activity was determined based on the decomposition of hydrogen peroxide (H_2_O_2_). The reaction was carried out in a cuvette containing 200 μL of potassium phosphate buffer (75 mmol L^−1^, pH 7.0), 90 μL of H_2_O_2_ (41.66 mmol L^−1^), and 10 μL of enzyme extract. The decrease in absorbance at 240 nm was monitored for 5 min, with readings taken every 15 s. Catalase activity was expressed as micromoles of H_2_O_2_ decomposed per minute per milliliter of extract.

### 2.5 Statistical Analyses

After verifying the assumptions of homogeneity of variances and normality of residuals, the water quality data were analyzed using Student's *t*‐test to compare influents versus effluents and ANOVA followed by Tukey's test to compare treatments. The COD removal kinetics were adjusted by first‐order regression with the residual (plateau), and the curves were compared using ANOVA. Agronomic variables (fresh and dry mass) were compared between the control and treated groups using Student's *t*‐test.

To evaluate antioxidant enzymes, specific approaches were adopted according to the nature of the data. For SOD, the value corresponding to 50% inhibition of activity was used as a comparative parameter between treatments, with differences evaluated using the *t*‐test. POX activity was determined based on the reaction rate calculated as the change in absorbance over time (ΔAbs/Δt), obtained from the slope of the linear portion of the absorbance versus time curve. The slope was calculated by linear regression using the initial reaction interval, ensuring high linearity, and the enzyme activity was expressed according to the variation in absorbance per unit time.

CAT activity was evaluated following the approach proposed by Román‐Ramos et al. (2024), in which enzymatic responses are integrated over time when reaction kinetics do not present linear behavior. Due to the instability of the absorbance–time curves and the low coefficients of determination obtained in the kinetic regressions, CAT activity was quantified as the area under the curve (AUC) of H_2_O_2_ consumption over the assay period. This method provides a robust estimate of cumulative enzymatic activity by integrating the total redox response, as supported by previous studies that demonstrate the validity of AUC‐based metrics for antioxidant enzymes under nonlinear kinetic conditions. The accumulated CAT activity was subsequently compared between control and treatment groups using Student's *t*‐test. Statistical analyses were conducted using an α level of 0.05. The *p*‐values between 0.05 and 0.10 were also reported, as they were considered indicative of statistical trends.

## 3 Results and Discussion

### 3.1 Phytoremediation

**FIGURE 2:**
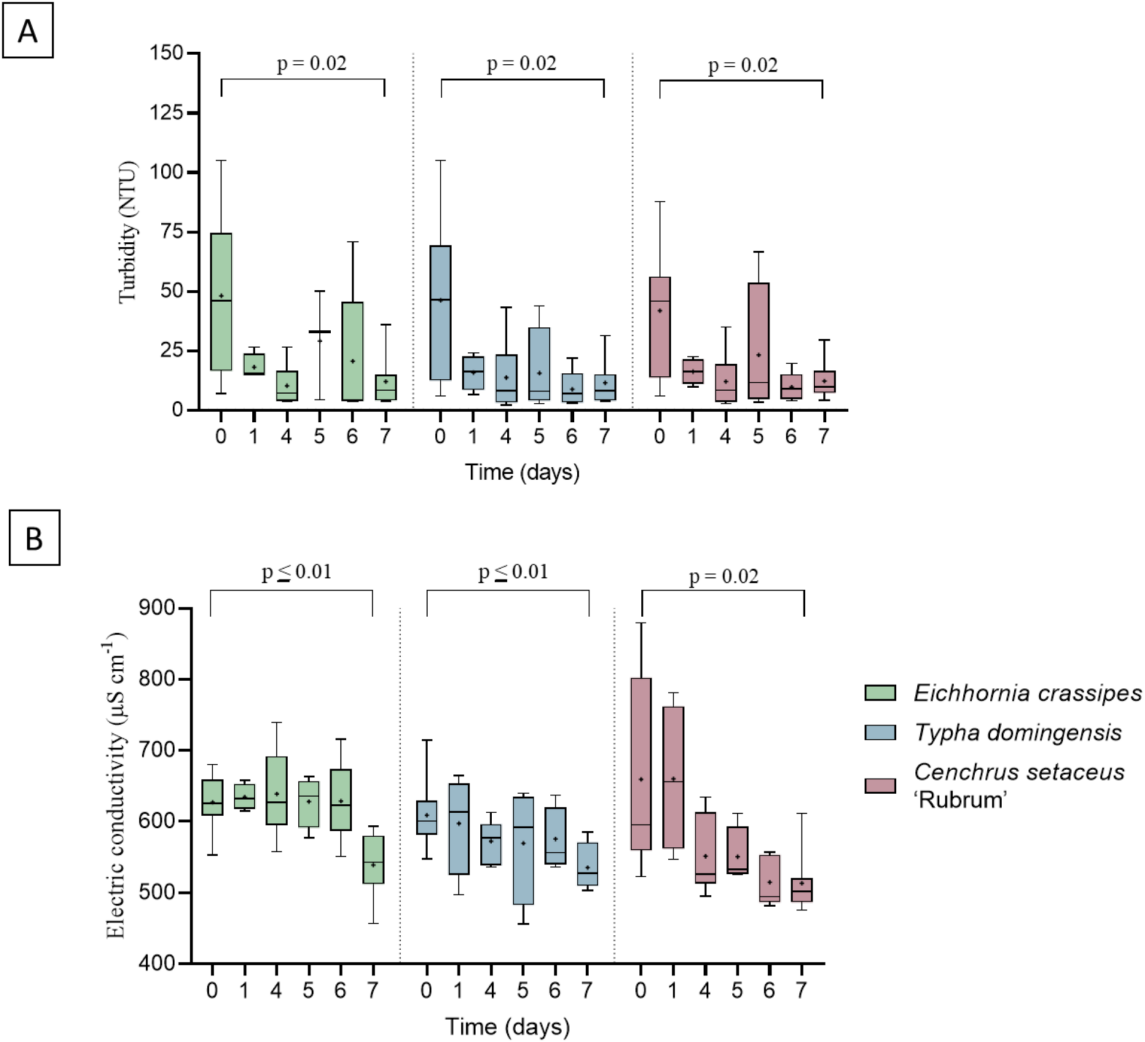
Daily variation of turbidity (A) and electrical conductivity (B) in the phytoremediation systems during the experimental batches. Values represent measurements of treated wastewater in hydroponic units cultivated with *Eichhornia crassipes* , *Typha domingensis* , and *Cenchrus setaceus* . Data correspond to the monitoring period of 70 days under sequential batch operation (7‐day hydraulic retention time per cycle). Statistical differences between influents and effluents were evaluated using Student's *t*‐test.

Figure 2 shows the average daily values of turbidity (A) and electrical condutivity (B) in the systems throughout the batches.

Turbidity, which was artificially increased by adding kaolin to ensure greater representativeness of suspended solids, decreased in the first few days, demonstrating the ability of the system to retain suspended particles. Despite the high variability attributed to the fluctuation of the particulate load (kaolin) in the influent, all treatments were effective in clarifying the water. Regarding dissolved solids, the electrical conductivity tended to decrease throughout the experimental cycles, with a significant difference for all plants. This decrease in the ionic load may be due to mechanisms such as absorption, phytoextraction, and salt retention by plants.

**FIGURE 3:**
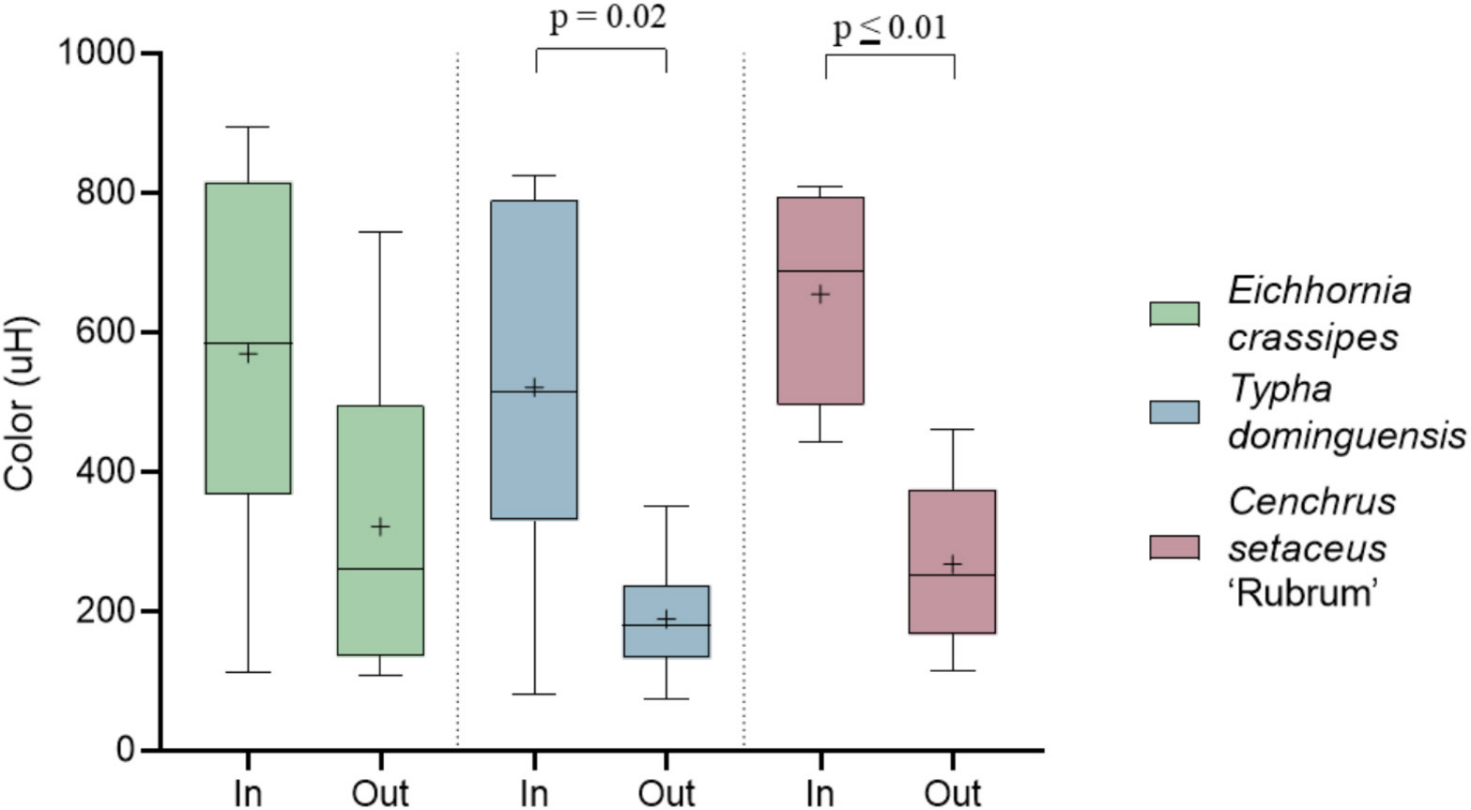
Color values in the systems on Day 0 and after 7 days of treatment. Statistical differences between influents and effluents were evaluated using Student's *t*‐test.

Figure 3 shows the color values throughout the experimental period.

*C. setaceus* showed the lowest *p*‐value between the influent and effluent compared to the other treatments, demonstrating high potential for removing substances responsible for discoloration. *T. domingensis* also demonstrated efficiency, with a significant reduction. In contrast, *E. crassipes* showed no statistical difference between the beginning and end of the experiment, indicating a lower capacity to remove coloration during the evaluated period.

**FIGURE 4:**
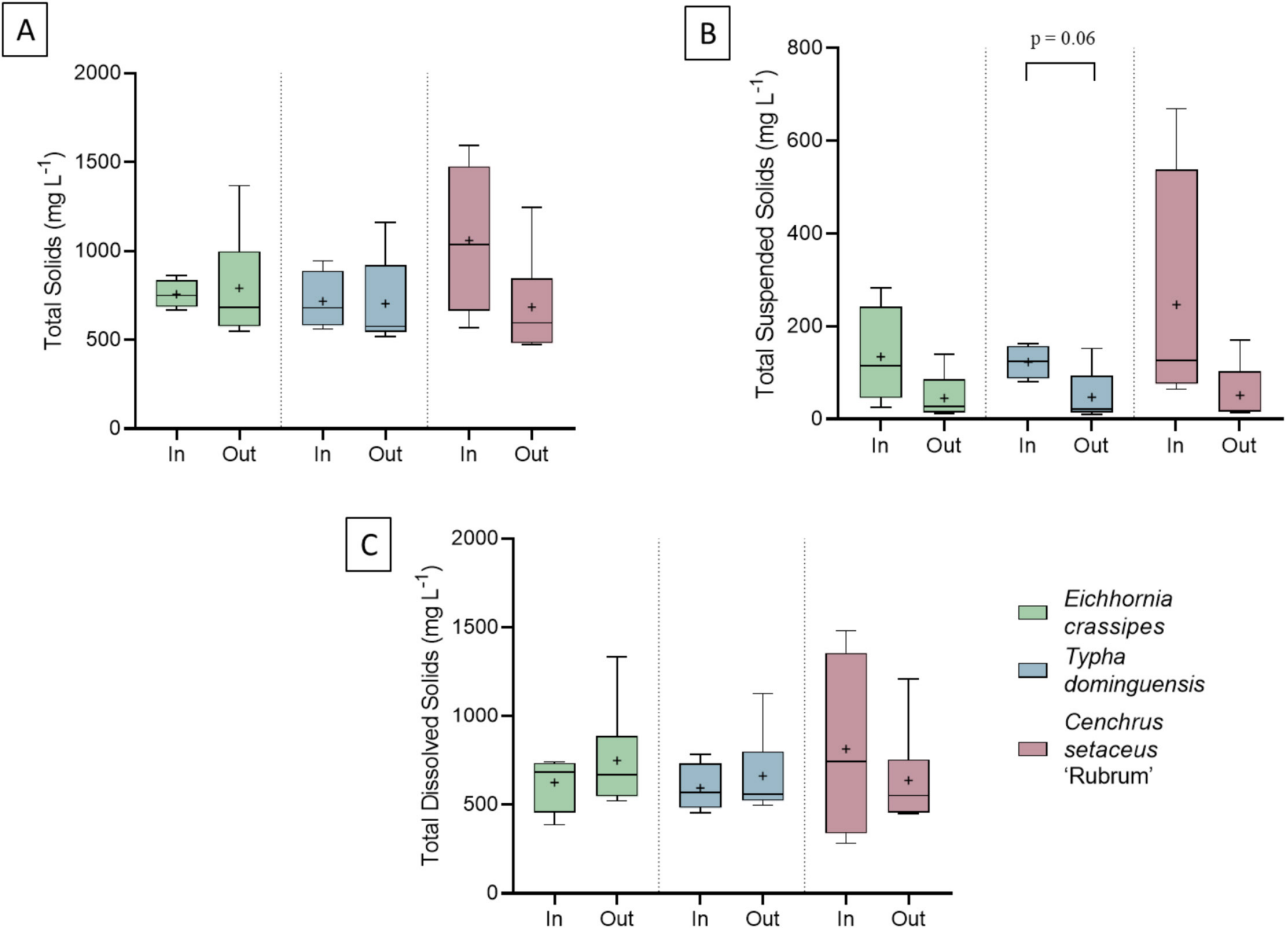
Influents and effluents concentrations for each treatment on Day 0 and after 7 days. (A) Total solids. (B) Total suspended solids. (C) Total dissolved solids. Statistical differences between influents and effluents were evaluated using Student's *t*‐test.

Figure 4 shows the concentration of the series of solids—total (A), total suspended (B), and total dissolved (C)—on Day 0 and after 7 days of contact between the wastewater and *E. crassipes* , *T. domingensis* , and *C. setaceus* .

In the evaluation of total solids (Figure 4A), both *E. crassipes* and *T. domingensis* showed slight variations in input and output values, with no clear trend toward removal. *C. setaceus* , despite having numerically lower median and mean values in the treated effluent, also did not show a significant difference.

Regarding total suspended solids (Figure 4B), although there was a narrowing of the interquartile range between days 0 and 7, indicating greater consistency in performance over time, the treatments with *E. crassipes* and *C. setaceus* did not show a statistically significant difference in TSS. This lack of significance is associated with the wide variation in influent values, which was directly influenced by the addition of kaolin, a source of fine particles that increased both suspended solids and turbidity in a nonhomogeneous manner throughout the experimental period. It should also be noted that, unlike turbidity (read daily), solids were measured weekly, which reduces the power of statistical tests in this case.

High variability in TSS is not uncommon in studies on macrophytes in treatment systems. Studies on constructed wetland systems have shown that the efficiency of suspended solids and turbidity removal varies widely depending on the nature of the influent, hydraulic conditions, and type of vegetation and that the removal of fine particles may be more dependent on physical processes (sedimentation and filtration) than isolated biological action, especially under conditions of high initial particulate load and additional support media for the physical retention of particles (Gomes et al. 2025).

For total dissolved solids (Figure 4C), although *C. setaceus* showed numerically lower mean values in the treated effluent, none of the treatments resulted in significant differences. However, as previously mentioned, in the treatments with *T. domingensis* and *C. setaceus* , there was a significant difference in color (*p* ≤ 0.01). This apparent discrepancy (significance in color and not in solids) may be related to the smaller amount of data available for solids (weekly) compared to color (daily) and to the fact that color was measured as apparent color, encompassing particulate matter, which can be read as turbidity (where significant removal was observed). Furthermore, in the case of *T. domingensis* , the significant reduction in total suspended solids supports the decrease in color associated with particulate matter, as these samples were not filtered for color analysis, indicating good physical retention efficiency. As already noted, for *C. setaceus* , although there was no statistical difference in solids, the high variability in the influent contributed to the dispersion of the data, even though the lower mean values in the effluent and the reduction in color suggest a greater removal potential compared to the other species.

**FIGURE 5:**
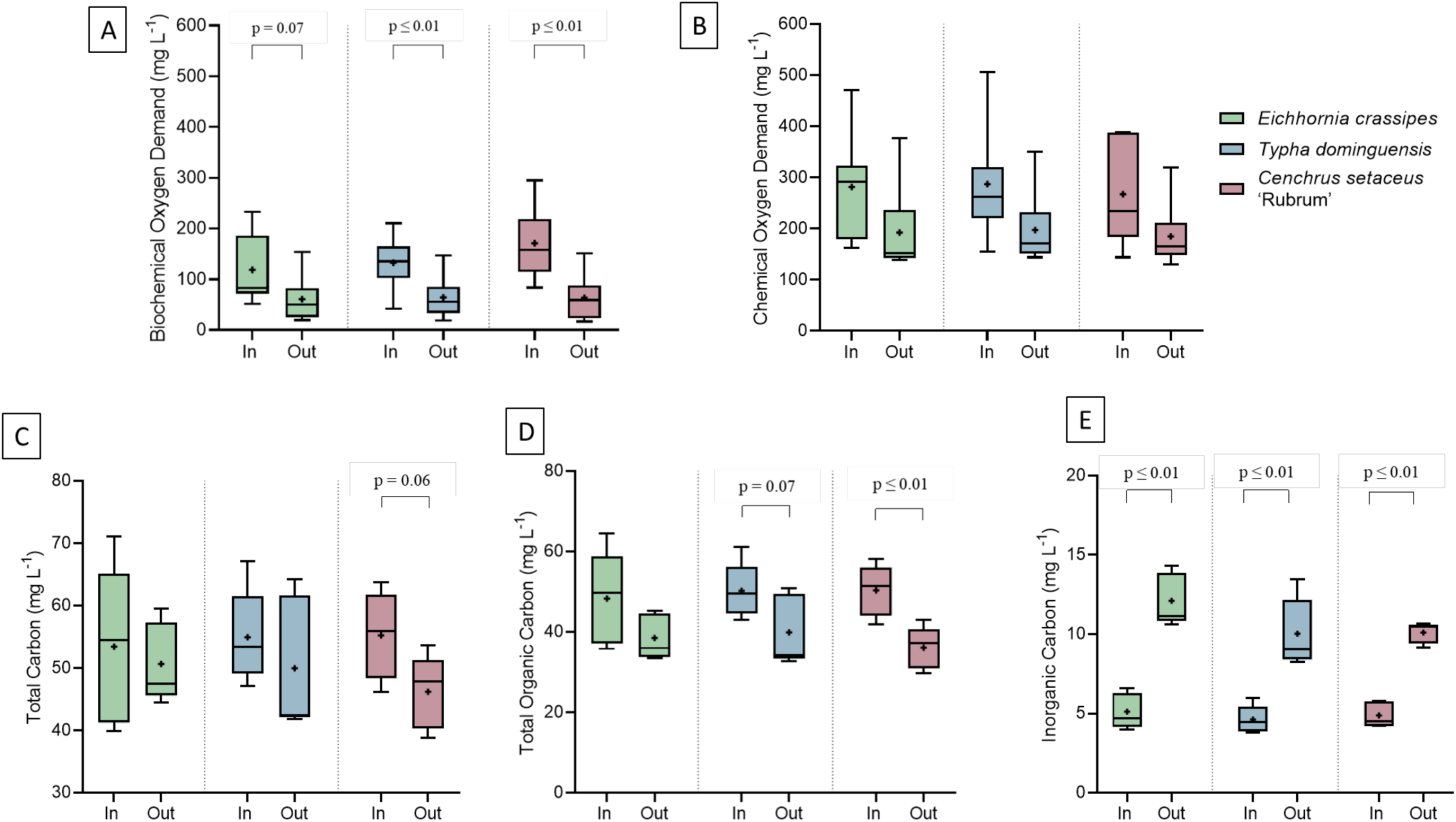
Influents and effluents concentrations for each treatment on Day 0 and after 7 days. (A) Biochemical oxygen demand. (B) Chemical oxygen demand. (C) Total carbon. (D) Total organic carbon. (E) Inorganic carbon. Statistical differences between influents and effluents were evaluated using Student's *t*‐test.

Figure 5 shows the data for the organic matter variables in the raw and treated samples for the different plant species.

Regarding COD, it was observed that none of the treatments promoted a statistically significant reduction (*p* > 0.10) according to the *t*‐test. The average numerical removal efficiency was 35.3% for *E. crassipes* , 40.5% for *T. domingensis* , and 48.2% for *C. setaceus* . Despite these differences, the mean final COD values between treatments were very similar, with no statistical difference between them according to Tukey's multiple comparison test (*p* > 0.10).

For BOD (Figure 5A), there were reductions between the influent and effluent values for all systems. In the *E. crassipes* , there was a significant reduction (*p* = 0.07), with an average efficiency of 48.8%. For units containing *T. domingensis* and *C. setaceus* , there were significant reductions (*p* ≤ 0.01), with efficiencies of 51.5% and 62.8%, respectively. However, as observed for COD, there was no difference between the treatments when compared to each other (Tukey test), indicating statistically equivalent performance, albeit with different numerical efficiencies compared to the influent.

All treatments reduced the BOD/COD ratio during contact with the plants. For the system with *E. crassipes* , the initial BOD/COD ratio was 0.41, which reduced to 0.32. For *T. domingensis* , the BOD/COD ratio decreased from 0.43 to 0.35. *C. setaceus* had an initial BOD/COD ratio of 0.53; after the treatment period, this ratio was reduced to 0.38, which represents the largest decrease among the treatments evaluated. This result accompanies the removal of BOD, suggesting a reasonable efficiency of the system containing *C. setaceus* in removing the biodegradable fraction of the organic load.

Regarding TOC, the system with *C. setaceus* showed a statistically significant reduction compared to its influent (*p* ≤ 0.01), indicating its ability to remove the dissolved organic fraction from the effluent. The unit with *T. domingensis* showed a reduction but with less statistical robustness (*p* = 0.07). As with the color, SST, and COD, the system cultivated with *E. crassipes* did not show a significant difference (*p* > 0.10), suggesting low effectiveness in TOC removal.

Considering that all species were cultivated in a hydroponic system, the differences observed in the removal of organic matter were not associated with the interaction between any packing material, but mainly with biological processes related to plant physiology and the mechanisms occurring in the rhizosphere. In this context, the lower efficiency of the system with *E. crassipes* may be associated with its photosynthetic rate and carbon allocation pattern.

Although *E. crassipes* is recognized for its rapid growth in eutrophic environments, its high photosynthetic rate tends to be directed mainly toward vegetative growth and accumulation of aboveground biomass, with a relatively lower contribution of root exudates. This lower carbon flux to the rhizosphere may limit the stimulation of heterotrophic microbial activity associated with roots, thereby reducing the degradation of dissolved organic matter (Maranho and Gomes 2024).

In the case of TC, which corresponded to the sum of the dissolved organic and inorganic fractions, only the system with *C. setaceus* showed a significant reduction between the influent and effluent, with a *p*‐value of 0.06. It is important to note that for the quantification of TC, the samples were previously filtered, which implies that the results refer exclusively to the dissolved fraction of carbon, disregarding the particulate fraction.

This methodological distinction is relevant for interpreting the results, especially when compared with COD and BOD data. COD measures the total load of organic matter (dissolved and particulate), whereas BOD is sensitive to the biodegradable fraction, which is generally more solubilized. Therefore, the absence of more robust statistical differences in COD, in contrast to the results for TOC, TC, and BOD, may be related to the fact that part of the persistent organic matter was in particulate form and not captured in the dissolved carbon analyses. Thus, the TC and BOD results indicated that much of the removal observed in the treatments occurred in the soluble phase.

The results for inorganic carbon (IC) (Figure 5E) showed a distinct behavior; in all treatments, there was an increase in the concentration of IC in the effluent, with these variations being statistically significant (*p* ≤ 0.01). This increase may be associated with root respiration and microbial activity, which release CO_2_ dissolved in water in the form of carbonic acid, carbonate, and bicarbonate or with the mineralization of organic matter, converting it into inorganic forms. This increase occurred in all three treatments, indicating that this phenomenon was common in all species evaluated. The mineralization of organic matter by microorganisms that oxidize organic compounds into CO_2_ contributes to the increase in IC in systems, as demonstrated by Clower et al. (2024) in the production of dissolved IC observed in natural wetlands, resulting from respiratory processes associated with the mineralization of organic matter. The fact that this increase occurred consistently in the three treatments indicates that this phenomenon of conversion of organic carbon to soluble inorganic forms is common to all species evaluated, emphasizing that even in vegetated and hydroponic systems, microbial and respiratory dynamics can be a dominant factor in the balance of dissolved IC.

The table shows the parameters of the first‐order curves with residual (plateau) (Equation 1), as well as the quality data of the adjustments of these models to the data. Data were collected on Days 0 (raw influent), 2, 4, 6, and 7 (treated effluent).

(1) $$C/C_{0}=\left(1-\mathrm{Rf}\right)\times \exp\;\left(-k\times t\right)+\mathrm{Rf}$$

where *C*_*0*_ is the COD concentration in the influent (mg L^−1^), *C* is the COD concentration at time *t* (mg L^−1^), *k* is the COD removal coefficient (d^−1^), and *Rf* (dimensionless) is the remaining fraction that is difficult to degrade (ratio between the residual concentration at the plateau and *C*_*0*_).

The first‐order model with residual provided an adequate description of COD decay in all systems (parameters in Table 1). Although the recalcitrant fraction (*Rf*) was similar across species, temporal dynamics differed: *C. setaceus* displayed the fastest removal (highest *k* and lowest *RMSE*), *T. domingensis* was intermediate, and *E. crassipes* the slowest. These kinetic differences indicate distinct degradation rates rather than differences in remaining recalcitrant COD and suggest that *C. setaceus* promotes faster organic matter turnover, likely through combined plant physiology and stronger plant–microbe interactions.

**TABLE 1:** Curve parameters and model fitting.

Curve parameters			
	*Eichhornia crassipes*	*Typha domingensis*	*Cenchrus setaceus*
*Rf*	0.58	0.55	0.57
*k* (d^−1^)	0.41	0.93	2.49

Adjustment parameters			
	*Eichhornia crassipes*	*Typha domingensis*	*C. setaceus*
*R* ^2^	0.52	0.65	0.67
*RMSE*	0.16	0.15	0.14

The decay curve of *C. setaceus* showed a marked reduction in COD concentration in the first days of contact, with a tendency to stabilize from the fourth day onwards. This indicates that much of the organic load removal occurred rapidly, suggesting that a HRT of less than 7 days could be sufficient to achieve good efficiency in this system.

The possibility of operating with a reduced HRT is highly advantageous in practical and economic terms, particularly in large‐scale applications. For example, it would be possible to reduce the volume of the reactor (smaller cultivation area), resulting in more compact structures and lower implementation and maintenance costs. The use of plants such as *C. setaceus* , which provide efficient removal in shorter periods, can therefore optimize system performance and promote high efficiency in less operating time.

In the study by Guarino et al. (2020), *C. setaceus* was evaluated for its phytoremediation capacity in TPH‐contaminated soils, with and without association with a consortium of plant growth‐promoting bacteria and arbuscular mycorrhizal fungi. The results indicated that when inoculated with the microbial consortium, 94% of TPH was removed from the soil, compared to 78% in treatments with non‐inoculated plants. Degradation was especially effective in the medium‐ and long‐chain aliphatic fractions (C13–C36). Accumulation of these compounds was observed in the roots (up to 45 mg kg^−1^) and to a lesser extent in the leaves (up to 19 mg kg^−1^) of *C. setaceus* , mainly in the inoculated systems. The greater removal in the inoculated systems was attributed to rhizodegradation, a process stimulated by the interaction between roots, plant exudates, and rhizosphere microbiota. This activity was confirmed by the increase in dehydrogenase activity in the soil, which is an indicator of microbial respiratory activity and, therefore, of the degradation of organic compounds.

**FIGURE 6:**
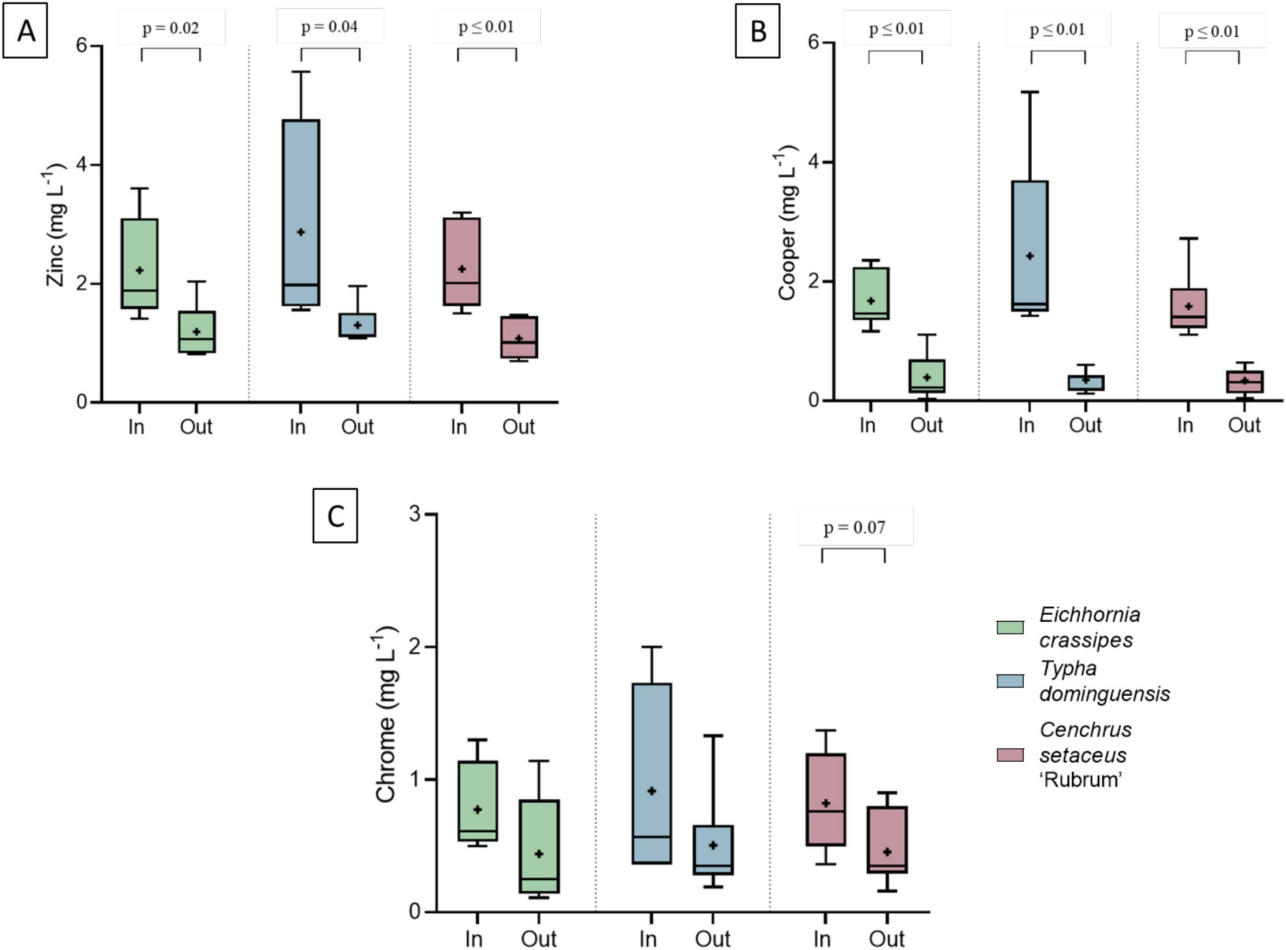
Effluent and influent concentration for each treatment on Day 0 and after 7 days. (A) Zinc. (B) Copper. (C) Chromium. Statistical differences between influents and effluents were evaluated using Student's *t*‐test.

Figure 6 shows the results of Zn, Cu, and Cr removal for the three species studied.

For zinc (Figure 6A), a significant reduction in concentrations was observed after treatment in all systems, with *p*‐values of 0.02, 0.04, and *p* ≤ 0.01 for *E. crassipes* , *T. domingensis* , and *C. setaceus* , respectively. After treatment, the average values in the effluents were 1.19 mg L^−1^ in *E. crassipes* , 1.30 mg L^−1^ in *T. domingensis* , and 1.08 mg L^−1^ in *C. setaceus* . *C. setaceus* and *T. domingensis* also had the lowest final minimum concentration (0.70 mg L^−1^), suggesting good efficiency and stability in the removal of this metal.

The zinc removal efficiencies were 46.3% for *E. crassipes* , 54.7% for *T. domingensis* , and 52.0% for *C. setaceus* . Although numerically the least efficient of the three, *E. crassipes* has been widely recognized for its ability to remove Zn from contaminated effluents. In studies by Nasir et al. (2022), this species demonstrated significant metal absorption, with reductions of up to 77.2 mg L^−1^ in 30 days of exposure, highlighting its phytoremediation potential.

These authors (Nasir et al. 2022) highlighted *E. crassipes* as an efficient accumulator of heavy metals such as zinc, with a high bioconcentration factor (BCF = 3862.7). This capacity is related to the presence of functional groups (C=S, C=N, and OH), chelating proteins and amino acids, such as cysteine, and the production of phytochelatins that aid in cellular detoxification. These mechanisms reinforce the potential for the phytoremediation of contaminated aquatic environments.

In the case of Cu (Figure 6B), all treatments showed significant differences between the influent and effluent. The average concentrations were reduced from 1.68 mg L^−1^ ( *E. crassipes* ), 2.43 mg L^−1^ ( *T. domingensis* ), and 1.58 mg L^−1^ ( *C. setaceus* ) to 0.39, 0.35, and 0.34 mg L^−1^, respectively. The lowest point values after treatment were observed in *E. crassipes* (0.03 mg L^−1^) and *C. setaceus* (0.04 mg L^−1^), indicating excellent performance of these systems. Mukhtar and Abdullahi (2017) highlighted the high bioconcentration potential of *T. domingensis* , with BCF values above 13 throughout the year, reaching 26.0, which indicates a significant capacity to absorb the metal directly from the water. In addition, the observed transfer factor (TF) values greater than 1 during part of the year suggest a good ability to translocate Cu from the sediment to the aerial part of the plant, which is an important characteristic for phytoextraction.

The removal of Cr (Figure 6C) was less significant. Only the system with *C. setaceus* showed a significant reduction between influent and effluent (*p* = 0.07). Taufikurahman et al. (2023) demonstrated that elephant grass ( *Pennisetum purpureum* ) removed more than 99% of Cr in 31 days in horizontal subsurface constructed wetland, highlighting mechanisms such as biosorption, root accumulation, and the reduction of Cr^+6^ to Cr^+3^. These results reinforce the potential of *C. setaceus* , which, although of different species, belong to the same genus and have morphological and physiological similarities. Although *C. setaceus* achieved 44.7% removal in just 7 days, it achieved higher efficiencies with longer retention times, given the similarity between the species.

The higher concentration factors observed for emergent species may also be related to their more developed root systems and greater rhizosphere interaction compared to floating species. Emergent macrophytes typically present larger root surface areas and stronger substrate contact, which can enhance pollutant adsorption and uptake processes.

Swaefy et al. (2020) demonstrated the high potential of *C. setaceus* “Rubrum” in the phytoremediation of cadmium (Cd) in contaminated soils, highlighting their effectiveness in both the absence and presence of chelating substances. Without the addition of citric acid, the species could remove 40%–60% of the Cd present in the soil. When associated with citric acid (1 mmol ^kg−1^), the performance was even more impressive, with removal exceeding 96%, starting from an initial Cd concentration of 100 mg kg^−1^ of Cd. TF values ranged from 1.17 to 1.66. These results are in line with the data from the present study, in which *C. setaceus* removed 44.7% of the Cr. Considering that removal mechanisms such as biosorption, complexation, and accumulation in biomass are common among different metals, the application of chelating agents, such as citric acid, may be a viable strategy to optimize chromium removal by this species.

The data confirm the potential of the three species in the phytoremediation of synthetic wastewater from oil well drilling. *C. setaceus* stood out as the most efficient, with significant reductions in BOD, TOC, and TC, in addition to the highest COD removal rate and the best fit to the kinetic model. Although all metals proved to be efficient, only *C. setaceus* showed a significant reduction in chromium.

### 3.2 Agronomic Performance

**FIGURE 7:**
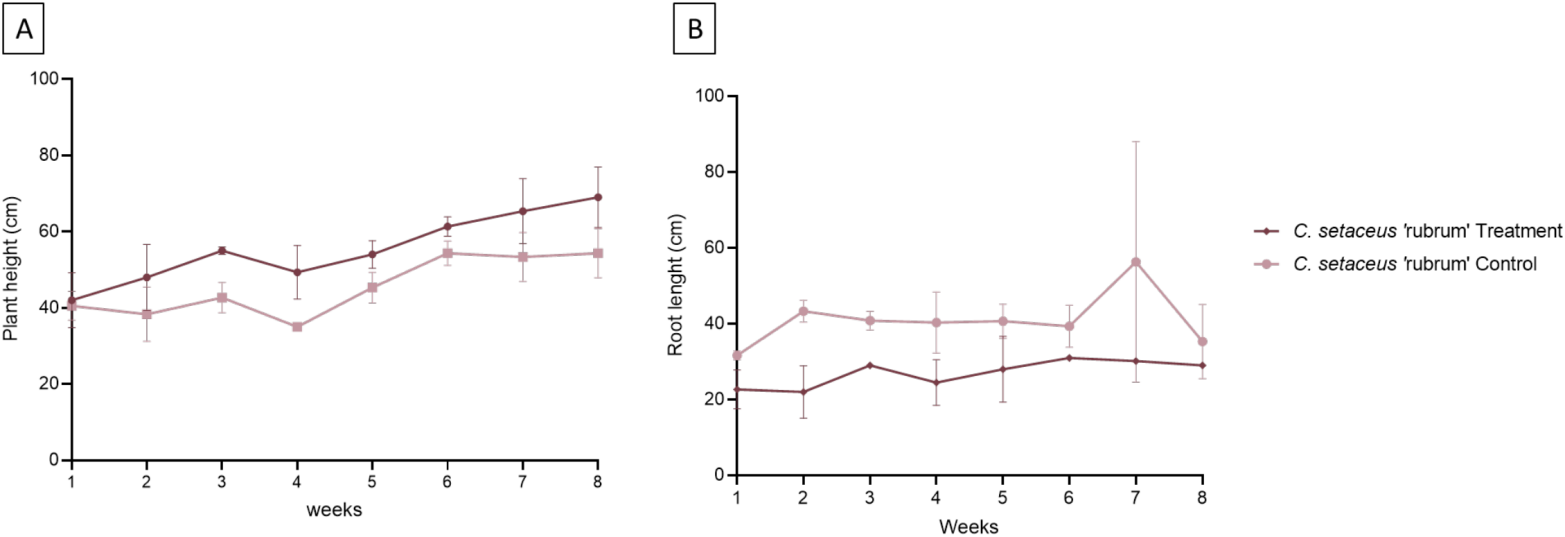
Height and root size as a function of monitoring weeks.

Figure 7 shows the agronomic performance of the plants evaluated over the weeks of monitoring, comparing two treatments: control (plants grown only with 15% Hoagland nutrient solution) and treatment system (plants exposed to synthetic effluent + 15% Hoagland solution). The variables monitored were the height of the aerial parts and the root length.

For the grasses *C. setaceus* , it was observed that treatment with effluent did not impair their height growth, maintaining a performance similar to that of the control over the weeks. However, in terms of root length, the grass showed a slight reduction when exposed to effluent. However, this difference was modest and did not visibly compromise the vigor of the plant, which was consistent with its outstanding efficiency in the treatment processes observed.

**FIGURE 8:**
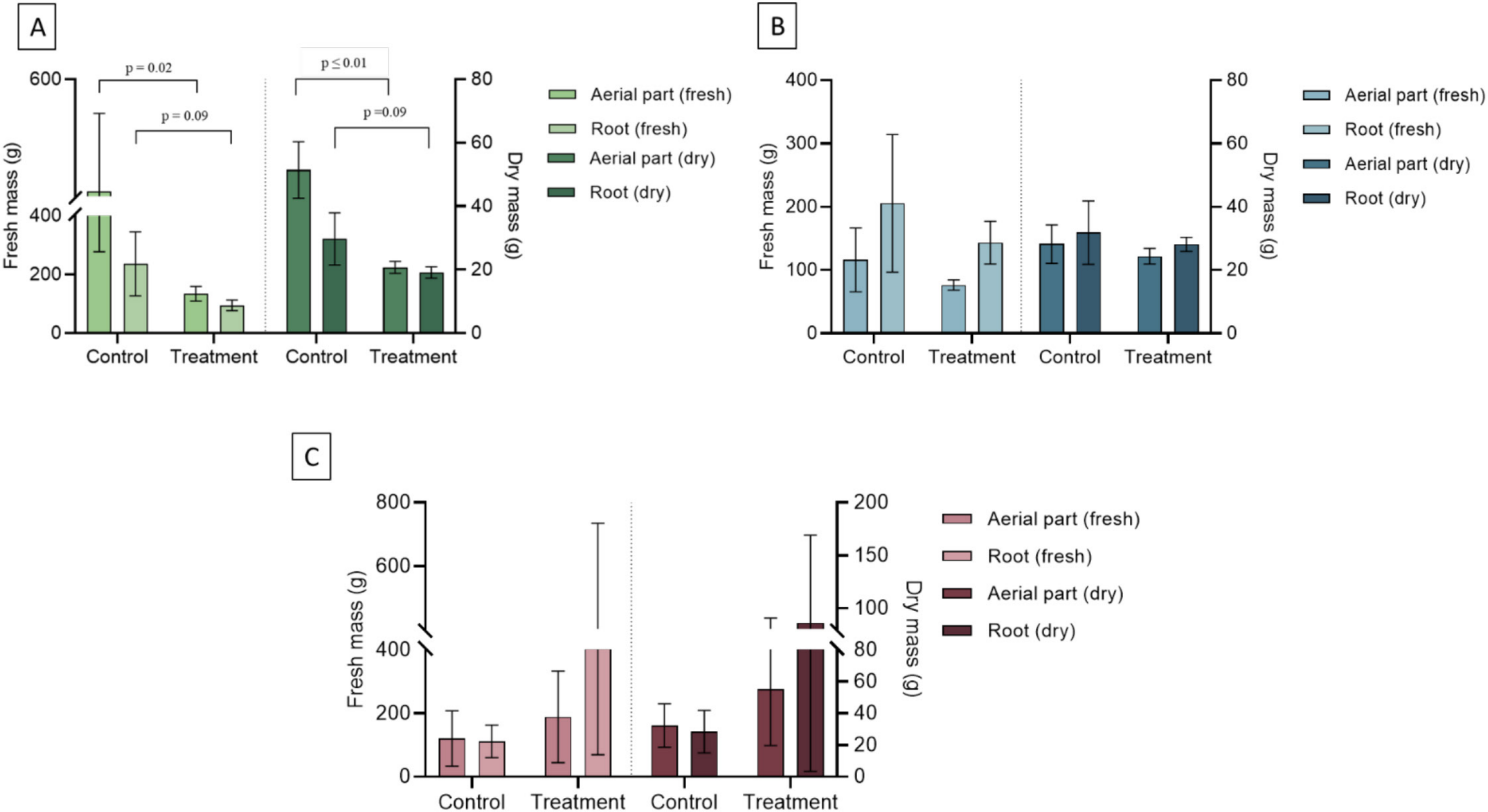
Fresh and dry biomass of roots and shoots of the plants after 70 days of operation. (A) *Eichhornia crassipes* . (B) *Typha domingensis* . (C) *Cenchrus setaceus* “Rubrum” after 70 days. Statistical differences between controls and treatments were evaluated using Student's *t*‐test.

When analyzing the results of fresh and dry mass of the roots and aerial parts of the three plant species after 70 days of cultivation, comparing the treatments with effluent and nutrient solution (control), it was observed that *C. setaceus* (Figure 8) showed stable agronomic performance. There were no significant differences between the treatments for any of the evaluated variables (fresh and dry mass of the aerial and root parts), which demonstrates the robustness of the species when exposed to effluent. In addition, no plants senesced during the experiment, which reinforces their tolerance and adaptability to environments with high contaminant load.

The proper disposal of plants used in phytoremediation processes is an essential step in ensuring the sustainability and environmental safety of these technologies. After fulfilling their role in removing pollutants such as metals, nutrients, or organic compounds, these plant biomasses often accumulate contaminants and cannot be disposed of in any way. In this context, the recovery of these plant wastes through strategies that allow for the recovery of resources is a promising alternative, in line with the principles of circular economy and the reuse of materials. Studies have highlighted that the post‐remediation management of contaminated biomass is a critical issue for the success of phytoremediation, requiring effective processing and reuse strategies to avoid environmental risks and take advantage of the resources accumulated in plants, including the recovery of metals and conversion into value‐added products such as biochar, biogas, and biofuels, in line with the principles of circular economy and sustainable agriculture (Mukherjee et al. 2025).

Recent studies indicate that thermochemical treatments such as pyrolysis may enhance the environmental safety of phytoremediation residues by reducing biomass volume and immobilizing accumulated pollutants in the biochar matrix. This process may also allow subsequent recovery of valuable elements from ash fractions, depending on contaminant concentration and economic feasibility. Therefore, biomass management strategies should be selected according to pollutant profile and regulatory requirements (Mukherjee et al. 2025; Tan et al. 2023).

### 3.3 Enzymatic Activity

**FIGURE 9:**
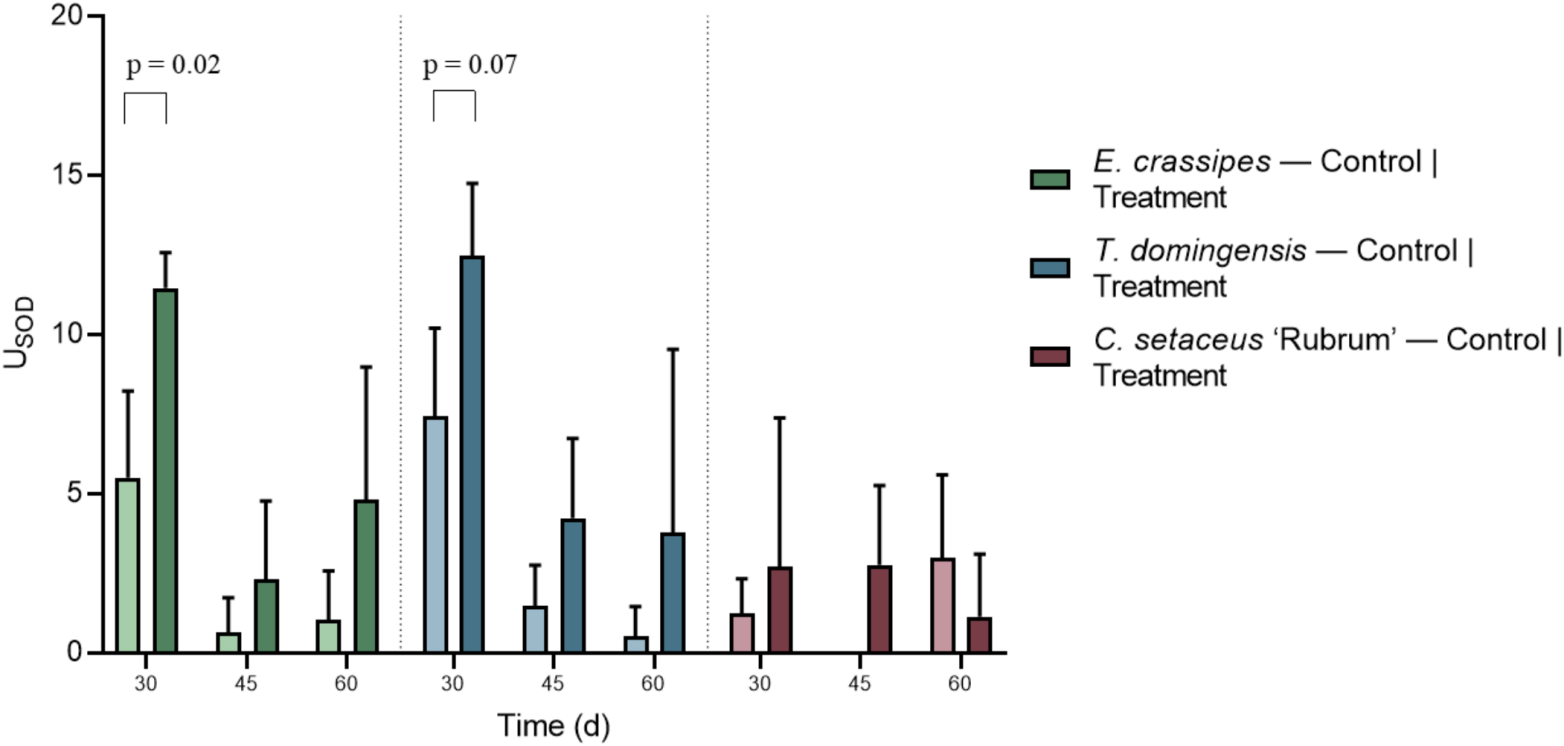
SOD unit: Amount of enzyme needed to inhibit 50% of superoxide radical formation in the analyzed reaction. Different letters differ by up to 10% by *t*‐test. Statistical differences between controls and treatments were evaluated using Student's *t*‐test.

Figure 9 shows the results obtained for SOD activity, highlighting the differences between the control (15% Hoagland nutrient solution) and those that received the synthetic effluent.

SOD activity was monitored throughout the experiment on Days 30, 45, and 60 after effluent application to assess the antioxidant response of the plant species to stress caused by the treatment. SOD is responsible for neutralizing superoxide radicals (O_2_^−^·) and is, therefore, a sensitive marker of oxidative stress.

In *E. crassipes* , SOD activity was higher in the effluent treatment on Day 30 (*p* = 0.02), indicating acute activation of the antioxidant system in response to the initial stress. On Days 45 and 60, although no statistically significant differences were observed between the treatment and control groups (*p* = 0.18 and 0.11, respectively), the values in the treated group remained numerically higher, suggesting a continuous response to oxidative stress, but with less intensity. The decline after the peak may indicate physiological adaptation over time, stabilization of antioxidant mechanisms, or even depletion of enzymatic response.

In *T. domingensis* , SOD activity was significantly higher in the effluent treatment on Days 30 and 45 (*p* = 0.07), indicating that the plant was under continuous oxidative stress during this period. These results are in line with physiological observations of the species, such as high growth variability and mortality of three individuals in Weeks 4 and 5, which highlights the sensitivity of *T. domingensis* to effluent exposure. However, as in *E. crassipes* , SOD response decreased on Day 60, which may be related to enzymatic depletion. Previous studies have reported that antioxidant enzyme activity may increase in the early stages of stress, followed by a decline in more advanced stages, reflecting the limitation or exhaustion of the enzymatic response under continuous oxidative stress conditions (Batool et al. 2022).

In contrast to these two species, *C. setaceus* showed the lowest SOD activity throughout the experiment. The absence of a significant response compared to the control, as well as the low levels of enzyme activity, suggests that *C. setaceus* has a higher tolerance to the stress generated by the effluent. This interpretation is consistent with the other results observed: No plants senesced during the 70 days of the experiment, and agronomic performance was superior.

Guarino et al. (2020) highlighted the importance of the microbial consortium in the response to oxidative stress caused by petroleum hydrocarbons in *C. setaceus* . In Group 1 (without consortium), SOD and CAT activity was higher than in the control, indicating stress, but lower than in Group 2 (with consortium), reflecting lower efficacy in neutralizing reactive oxygen species and, therefore, lower efficiency in phytoremediation. In Group 2, microbial inoculation increased the activities of these enzymes, demonstrating that microorganisms enhance the cellular defense mechanisms of plants, promote their growth, protect plant tissues, and optimize the removal of pollutants.

**FIGURE 10:**
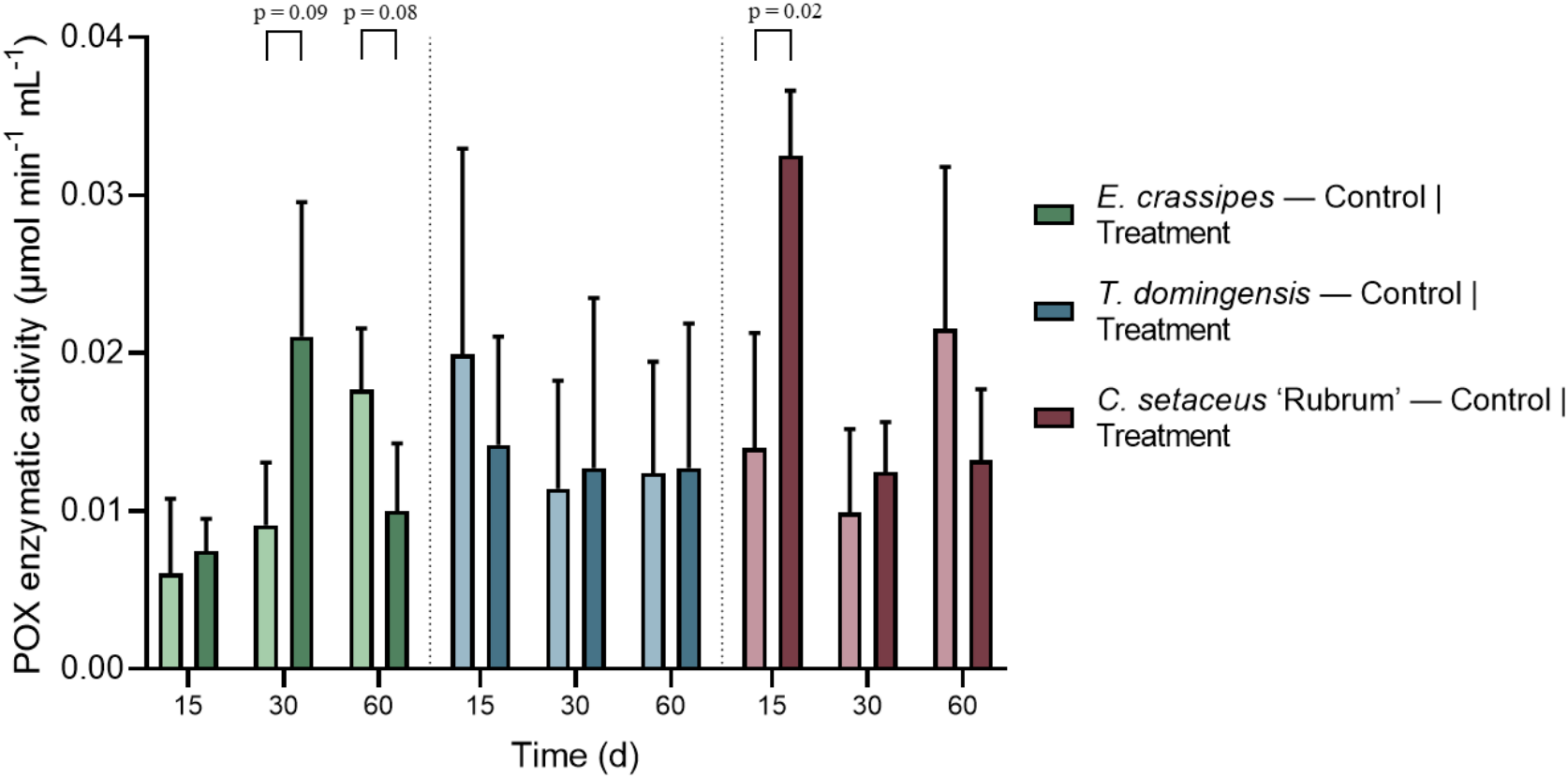
Peroxidase (POX) activity of the three studied plant species measured at 15, 30, and 60 days after the beginning of effluent exposure, expressed as μmol min^−1^ mL^−1^. Statistical differences between controls and treatments were evaluated using Student's *t*‐test.

Figure 10 presents the peroxidase activity of the three studied plant species at 15, 30, and 60 days after the beginning of exposure to the effluent, allowing the comparison between control and treatment groups over time.

For *E. crassipes* , POX activity exhibited a time‐dependent response to the effluent exposure. No statistically significant difference between control and treatment was observed on Day 15 (*p* > 0.1), indicating that at the early stage of exposure, the antioxidant system of the plants had not yet been markedly differentiated by the presence of the effluent.

A significant difference emerged on Day 30 (*p* = 0.09), with the treatment group showing higher POX activity than the control. This peak in enzymatic activity likely reflects a strong activation of the antioxidant defense system in response to the accumulation of toxic compounds from the effluent, such as metals and hydrocarbons. At this stage, POX appears to play a key role in mitigating oxidative damage, suggesting that Day 30 represents a critical point of oxidative stress for *E. crassipes* under treatment conditions.

By Day 60, the pattern was reversed, with the control group exhibiting significantly higher POX activity than the treated plants (*p* = 0.08). This decline in POX activity in the treatment group may indicate metabolic exhaustion or a reduced capacity to sustain antioxidant defenses after prolonged exposure to the effluent. Such a response suggests that, despite the initial activation of protective mechanisms, *E. crassipes* may experience physiological overload over time, leading to impaired redox homeostasis under chronic stress conditions.

Additionally, an overall increase in POX activity over time (15, 30, and 60 days) was observed in both control and treatment groups. This trend may be partially attributed to plant aging and the short life cycle characteristic of *E. crassipes* . As plants progress through their developmental stages, age‐related metabolic adjustments and senescence‐associated oxidative processes can naturally elevate antioxidant enzyme activity. Therefore, the temporal increase in POX activity likely reflects the combined effects of ontogenetic development and environmental stress, rather than effluent exposure alone (Bartoli et al. 1995).

For *T. domingensis* , no statistically significant differences were observed between the control and treatment groups at any of the evaluated time points (*p* > 0.1). This lack of statistical distinction suggests a limited capacity of *T. domingensis* to modulate peroxidase activity in response to the imposed stress. Consequently, POX does not appear to be a sensitive or effective biomarker of oxidative adjustment for this species under the experimental conditions, which may partially explain the occurrence of plant mortality observed during the experiment.

*C. setaceus* exhibited a significant difference between the treatment and control groups at Day 15, with the treatment showing higher POX activity (*p* = 0.02). This response indicates an early and transient activation of the antioxidant defense system upon initial exposure to the effluent. This increase can be interpreted not as a negative indication of damage but rather as an adaptive and effective response by the plant. The increase in POX activity demonstrated that the species proactively activated its antioxidant pathway to combat the reactive oxygen species generated in response to the effluent (Tanwir et al. 2021). This ability to efficiently mobilize and sustain its antioxidant defense mechanisms, without showing mortality or visible signs of agronomic stress, reinforces the high potential of *C. setaceus* as a stress‐tolerant species capable of maintaining its vitality and functions even in stressful environments.

The activation of POX can be understood through the composition of the synthetic effluent, which contained metals (Zn, Cu, and Cr) and hydrocarbons (hexane, hexadecane, and naphthalene). Heavy metals can replace essential metal ions in enzymes, alter the permeability of cell membranes, and catalyze Fenton reactions, leading to the production of highly toxic free radicals (Mansoor et al. 2023). Chromium, in particular when present in the hexavalent form, is extremely reactive and can generate high levels of ROS (Wakeel et al. 2020). Copper, although an essential micronutrient, disrupts redox homeostasis, and zinc interferes with enzyme activity and membrane integrity at high concentrations (Štolfa et al. 2015).

The absence of significant differences at later stages, combined with the lack of visible stress symptoms and zero plant mortality, indicates that *C. setaceus* successfully achieved physiological acclimation. In this context, the normalization of POX activity over time may reflect reduced oxidative pressure or efficient integration of detoxification mechanisms, rather than exhaustion of antioxidant capacity. This ability to respond quickly and subsequently maintain metabolic stability is a highly desirable trait in phytoremediation systems.

Reviews and comparative studies show that tolerant genotypes/species often display a prompt but short‐lived rise in enzymatic antioxidants followed by stabilization, whereas sensitive ones may either fail to activate or exhaust defenses later. *C. setaceus* pattern is therefore consistent with an adaptive response that limits long‐term damage (Wang et al. 2019).

**FIGURE 11:**
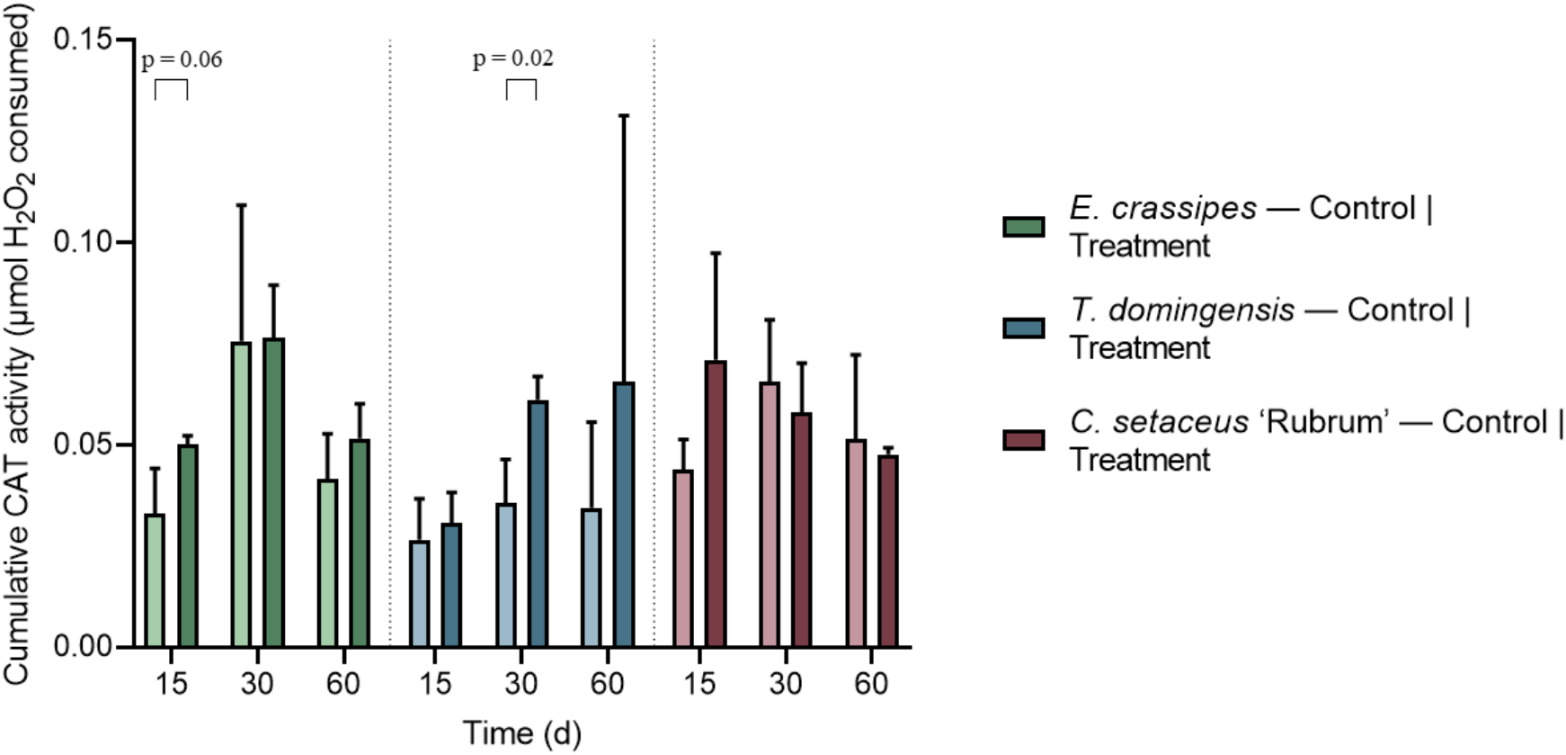
Cumulative catalase (CAT) activity of the three studied plant species at 15, 30, and 60 days after the beginning of effluent exposure. Statistical differences between controls and treatments were evaluated using Student's *t*‐test.

Figure 11 shows the cumulative catalase activity, expressed as the area under the absorbance–time curve (AUC), highlighting differences in antioxidant response dynamics among species and sampling periods.

Due to the high variability observed in the CAT kinetic readings, reflected by low coefficients of determination (*R* ^2^) in the absorbance–time regressions, cumulative CAT activity was evaluated using the AUC. This approach integrates the total enzymatic response over the assay period and provides a more robust representation of antioxidant performance when instantaneous reaction rates are unstable.

The temporal variation in accumulated CAT activity in *E. crassipes* indicates that the observed pattern is largely driven by the interaction between an early stress response and ontogenetic processes associated with plant aging and senescence. On Day 15, higher CAT values in the treatment compared to the control suggest an initial antioxidant response to the effluent (*p* = 0.06), reflecting increased H_2_O_2_ detoxification under early oxidative stress. This response is consistent with the high physiological plasticity of *E. crassipes* , which is known to rapidly activate antioxidant mechanisms under chemical stress.

By Day 30, a marked increase in CAT activity was observed in both control and treatment groups, with reduced differences between them. This pattern indicates that the elevated CAT activity at this stage is not exclusively related to effluent exposure but is also strongly influenced by intrinsic developmental processes. Age‐related increases in respiratory activity and basal reactive oxygen species production are known to enhance antioxidant enzyme activity, particularly in fast‐growing aquatic plants, leading to a convergence of CAT responses between experimental conditions.

At Day 60, the decline in accumulated CAT activity suggests metabolic exhaustion and the onset of senescence, which is compatible with the short life cycle of *E. crassipes* . During senescence, enzymatic antioxidant capacity often decreases due to protein degradation and reduced metabolic efficiency, even if oxidative pressure persists. Therefore, the late reduction in CAT activity likely reflects physiological aging rather than an adaptive response to the effluent.

The cumulative CAT activity showed species‐ and time‐dependent patterns, indicating differences in the capacity to detoxify hydrogen peroxide under effluent exposure. For *T. domingensis* , a statistically significant difference between control and treatment was observed only at 30 days (*p* = 0.02), with higher cumulative CAT activity in the treated plants. This result suggests an increased demand for H_2_O_2_ detoxification at this intermediate stage of exposure, likely associated with the accumulation of oxidative stress generated by effluent constituents.

The use of AUC proved appropriate for CAT evaluation in this study, as it captures the integrated antioxidant response and avoids overinterpretation of noisy kinetic data. From a physiological perspective, the cumulative CAT activity highlights the capacity of plants to maintain hydrogen peroxide detoxification over time, offering a reliable metric to compare oxidative stress management among species under challenging environmental conditions.

For *C. setaceus* , no statistically significant differences in accumulated CAT activity were observed between the control and treatment groups at any of the evaluated time points. Nevertheless, on Day 15, the treatment showed a higher mean CAT value than the control, suggesting a transient activation of the antioxidant system in response to the initial exposure to the effluent. In the subsequent collections, CAT activity progressively decreased in both groups, indicating attenuation of the oxidative response over time. This pattern suggests efficient physiological regulation and metabolic stabilization, with no evidence of sustained oxidative stress or antioxidant overactivation throughout the experimental period.

Hydrocarbons, hexane, hexadecane, and naphthalene are recalcitrant and hydrophobic compounds that accumulate in cell membranes, destabilizing their structures and causing lipid peroxidation. Plant metabolism of these compounds is limited, and their presence can affect processes such as photosynthesis, nutrient transport, and cell division (Janbazi et al. 2024). Under these adverse conditions, POX and CAT activation is an adaptive response that limits the damage caused by environmental stressors.

González et al. (2015) found in their study that *E. crassipes* at low Cr concentrations immediately induced SOD/CAT/POX, quickly restoring the redox balance and maintaining growth. The authors concluded that *E. crassipes* can grow under Cr^+3^ precisely because it increases the antioxidant defense. Similarly, in studies by González et al. (2018), under Zn stress (2–9 ppm), *E. crassipes* increased enzymatic activity without increasing lipoperoxidation (MDA), suggesting a protective effect of Zn in activating antioxidants. In other macrophytes, such as *Typha latifolia* , *Phragmites australis* , and *Typha angustifolia* , a similar pattern was observed: Exposure to metal mixtures increased all measured antioxidant enzymes (SOD, CAT, POX, and GR) in the roots and leaves (Schröder et al. 2009). For example, *Typha* exposed to Cd and As increased total peroxidase and glutathione reductase activity, indicating a reallocation of resources for antioxidant defense. These results show that *E. crassipes* and *T. domingensis* activate their antioxidant systems to tolerate metals and continue to remove contaminants from water.

Grass species also exhibit strong enzymatic induction in response to metals. In *Pennisetum*, plants subjected to Cr^+6^ accumulated metal mainly in the roots and maintained growth despite toxicity owing to increased SOD, POX, and CAT (Ram et al. 2019). Even with the addition of a chelating agent (EDTA), the combination of elevated antioxidants indicated resistance to Cr^+3^ and enabled phytostabilization in the soil (Ram et al. 2019). In fast‐growing grasses (such as *C. setaceus* or Brachiaria and similar species), an equivalent response is expected: High biomass combined with intense antioxidant activity favors metal extraction or phytostabilization.

Enzyme activity analysis showed distinct responses to chemical stress among the species. *E. crassipes* showed peak SOD and POX levels on Day 30, followed by a decline in subsequent collections, possibly related to the exhaustion of the antioxidant response, reflected in the death of the two plants. *T. domingensis* activated SOD, CAT, and POX at the beginning, but lost performance over time, with a decrease in antioxidant activity and mortality of individuals, indicating a low tolerance to effluent. *C. setaceus* showed a balanced enzymatic response: stable SOD, efficient CAT, and increased POX in the treatment, with no mortality and good development, indicating a greater capacity to adapt to stress. These results reinforce that *C. setaceus* is the most tolerant and promising species for use in vegetated treatment systems.

## 4 Conclusions

This study identified *C. setaceus* as a promising species for application in wastewater treatment from oil well drilling. This plant species stood out for its high agronomic resilience, absence of senescence, good vegetative development, and satisfactory performance in pollutant removal, with values of 48.2% for COD and significant reductions in heavy metals. In addition, it exhibited the fastest organic matter degradation kinetics among the evaluated species. Analysis of antioxidant enzyme activity (SOD, CAT, and POX) revealed a consistent biochemical response to chemical stress, unlike *E. crassipes* and *T. domingensis* , which showed metabolic instability and senescence during the experiment.

To reduce the interference of uncontrollable variables present in real effluents, which have a highly complex and variable composition, a synthetic effluent with key contaminants (Zn, Cu, Cr, hexane, hexadecane, and naphthalene) was used, allowing for a more accurate assessment of the tolerance and responsiveness of each plant. The selection of *C. setaceus* under these controlled conditions may represent an essential step for future tests with real wastewater‐ and nature‐based systems, expanding its potential for full‐scale applications.

The findings of this study have important implications for practical applications in constructed wetland systems designed to treat industrial oil waste. The high physiological tolerance and consistent performance of *C. setaceus* suggest that this species can sustain long periods of operation under high contaminant loads, thereby reducing the risk of system collapse due to plant stress. Additionally, the faster kinetics of organic matter removal and its efficacy in metal retention, especially chromium, indicate the potential for reducing HRT, and consequently, the costs and area required for system implementation. Thus, the incorporation of *C. setaceus* in constructed wetlands may represent a technically viable and environmentally sustainable alternative, contributing to the development of more efficient and resilient treatment strategies that are aligned with the principles of circular economy and integrated industrial waste management.

This study was conducted at pilot scale using 40‐L hydroponic reservoirs in a greenhouse environment, which imposed limitations on experimental replication, with one reactor per treatment containing three plants. Although multiple operational cycles were monitored, the limited number of reactor units may reduce statistical power for parameters influenced by high influent variability, such as COD. Although COD removal did not reach statistical significance, significant differences were observed for other parameters, including BOD and TOC. In addition, enzymatic analyses were conducted with a limited number of biological samples, which should be considered when interpreting physiological responses. Therefore, the differences observed among plant species should be interpreted as indicative performance trends under the tested conditions. Future studies with larger replicated systems are recommended to confirm these findings and further explore the mechanisms involved in pollutant removal and plant physiological responses.

## Author Contributions

**Thiago Oliveira de Souza:** conceptualization, methodology, formal analysis, investigation, data curation, writing – original draft, validation. **Tiago Antônio de Oliveira Mendes:** conceptualization, writing – review and editing, supervision, formal analysis. **Larissa Cassemiro Pacheco Monteiro:** methodology, investigation, writing – review and editing, formal analysis. **Luciano Endrigo Watthier Júnior:** investigation, methodology, formal analysis, writing – review and editing. **Diego da Silva Marques:** methodology, investigation, formal analysis, writing – review and editing. **Alisson Carraro Borges:** conceptualization, funding acquisition, writing – review and editing, formal analysis, project administration, supervision, resources.

## Conflicts of Interest

The authors declare no conflicts of interest.

## Data Availability

The data that support the findings of this study are available from the corresponding author upon reasonable request.

## References

[wer70385-bib-0001] Agnihotri, M. , U. Bhan , V. R. Nagalakshmi , et al. 2023. “Environmental Impact of Drilling Fluid Waste and Its Mitigation Techniques.” 101–112. 10.1007/978-981-99-2870-5_13.

[wer70385-bib-0002] Ahsan, M. 2024. “Fluid Materials: An ISO14000 Analysis Focusing on Material Impact.” International Journal of Science and Research Archive 13, no. 2: 3940–3943. 10.30574/ijsra.2024.13.2.2663.

[wer70385-bib-0003] Amakiri, K. T. , A. R. Canon , M. Molinari , and A. Angelis‐Dimakis . 2022. “Review of Oilfield Produced Water Treatment Technologies.” Chemosphere 298: 134064. 10.1016/j.chemosphere.2022.134064. 35240151

[wer70385-bib-0004] APHA (American Public Health Association) , AWWA (American Water Works Association) , and WEF (Water Environment Federation) . 2023. Standard Methods for the Examination of Water and Wastewater. 24th ed. American Public Health Association.

[wer70385-bib-0005] Bartoli, C. G. , M. Simontacchi , J. J. Guiamet , E. Montaldi , and S. Puntarulo . 1995. “Antioxidant Enzymes and Lipid Peroxidation During Aging of *Chrysanthemum morifolium* RAM Petals.” Plant Science 104, no. 2: 161–168. 10.1016/0168-9452(94)04020-H.

[wer70385-bib-0006] Batool, R. , M. J. Umer , B. Hussain , M. Anees , and Z. Wang . 2022. “Molecular Mechanisms of Superoxide Dismutase (SODs)‐Mediated Defense in Controlling Oxidative Stress in Plants.” In Antioxidant Defense in Plants, 157–179. Springer Nature Singapore. 10.1007/978-981-16-7981-0_8.

[wer70385-bib-0007] Borges, C. V. , R. O. Orsi , M. Maraschin , and G. P. P. Lima . 2023. “Oxidative Stress in Plants and the Biochemical Response Mechanisms.” In Plant Stress Mitigators, 455–468. Elsevier. 10.1016/B978-0-323-89871-3.00022-7.

[wer70385-bib-0008] Clower, P. O. , K. Maiti , and M. Bowles . 2024. “Contrasting Organic Carbon Respiration Pathways in Coastal Wetlands Undergoing Accelerated Sea Level Changes.” Science of the Total Environment 949: 174898. 10.1016/j.scitotenv.2024.174898. 39059644

[wer70385-bib-0009] Deville, J. P. 2022. “Drilling Fluids.” In Fluid Chemistry, Drilling and Completion, 115–185. Elsevier. 10.1016/B978-0-12-822721-3.00010-1.

[wer70385-bib-0010] Gomes, P. C. S. , I. S. P. Rochinha , J. N. A. de Oliveira , et al. 2025. “Effects of Plant and Substrate Types on Turbidity Removal in Constructed Wetlands: Experimental and w‐C* Model Validation.” Water (Basel) 17, no. 13: 1921. 10.3390/w17131921.

[wer70385-bib-0011] González, C. I. , M. A. Maine , J. Cazenave , G. C. Sanchez , and M. P. Benavides . 2015. “Physiological and Biochemical Responses of *Eichhornia crassipes* Exposed to Cr (III).” Environmental Science and Pollution Research 22, no. 5: 3739–3747. 10.1007/s11356-014-3558-4. 25263412

[wer70385-bib-0012] González, C. I. , M. A. Maine , H. R. Hadad , G. C. Sanchez , M. P. Benavides , and M. A. Campagnoli . 2018. “Effects on *Eichhornia crassipes* Under Zn Stress.” Environmental Science and Pollution Research 25, no. 27: 26957–26964. 10.1007/s11356-018-2741-4. 30008163

[wer70385-bib-0013] Gonzalez‐Flo, E. , X. Romero , and J. García . 2023. “Nature Based‐Solutions for Water Reuse: 20 Years of Performance Evaluation of a Full‐Scale Constructed Wetland System.” Ecological Engineering 188: 106876. 10.1016/j.ecoleng.2022.106876.

[wer70385-bib-0014] Guarino, C. , M. Marziano , M. Tartaglia , et al. 2020. “Poaceae With PGPR Bacteria and Arbuscular Mycorrhizae Partnerships as a Model System for Plant Microbiome Manipulation for Phytoremediation of Petroleum Hydrocarbons Contaminated Agricultural Soils.” Agronomy 10, no. 4: 547. 10.3390/agronomy10040547.

[wer70385-bib-0015] Ismail, N. , and A. Talebi . 2023. “Phytoremediation Approaches for Organic Pollutants.” In Bioremediation Technologies, 165–176. De Gruyter. 10.1515/9783111016825-009.

[wer70385-bib-0016] Janbazi, Z. , F. Zarinkamar , and S. Mohsenzadeh . 2024. Exploring the Phytoremediation Capacity of *Portulaca oleracea* Naphthalene Aromatic Hydrocarbon Contaminants: A Physiological and Biochemical Study. Springer Science and Business Media LLC. 10.21203/rs.3.rs-3950051/v1. 39256335

[wer70385-bib-0017] Khavari‐Nejad, s. 2019. “A Review on Plant Peroxidases.” Nova Biologica Reperta 5, no. 4: 428–437. 10.29252/nbr.5.4.428.

[wer70385-bib-0018] Mansoor, S. , A. Ali , N. Kour , et al. 2023. “Heavy Metal Induced Oxidative Stress Mitigation and ROS Scavenging in Plants.” Plants 12, no. 16: 3003. 10.3390/plants12163003. 37631213 PMC10459657

[wer70385-bib-0019] Maranho, L. T. , and M. P. Gomes . 2024. “Morphophysiological Adaptations of Aquatic Macrophytes in Wetland‐Based Sewage Treatment Systems: Strategies for Resilience and Efficiency Under Environmental Stress.” Plants 13, no. 20: 2870. 10.3390/plants13202870. 39458817 PMC11511398

[wer70385-bib-0020] Marinho, L. S. , B. C. Pereira , F. P. Guandalim , and L. M. Cavalcante . 2024. “Monitoring of Drilling Fluids and Cuttings as an Environmental Management Tool for Offshore Fluid Operations.” May 9, 2024. 10.4043/35329-MS.

[wer70385-bib-0021] Mukherjee, S. , A. C. Leri , C. Bandaranayaka , et al. 2025. “Sustainable Management of Post‐Phytoremediation Biomass.” Energy, Ecology and Environment 10, no. 6: 675–709. 10.1007/s40974-025-00364-w.

[wer70385-bib-0022] Mukhtar, A. A. , and I. L. Abdullahi . 2017. “Heavy Metals Phytoremediation Using *Typha domingensis* Flourishing in an Industrial Effluent Drainage in Kano, Nigeria.” Bayero Journal of Pure and Applied Sciences 10, no. 1: 277–280. 10.4314/bajopas.v10i1.41.

[wer70385-bib-0023] Mushtaq, N. U. , S. Saleem , A. Rasool , et al. 2022. “Functional Characterization of the Antioxidant Enzymes in Plants Exposed to Environmental Stresses.” In Antioxidant Defense in Plants, 15–30. Springer Nature Singapore. 10.1007/978-981-16-7981-0_2.

[wer70385-bib-0024] Nasir, M. , M. Nur , D. Pandiangan , et al. 2022. “Phytoremediation Study of Water Hyacinth (Eichhornia Crassipes) on Zinc Metal Ion (Zn2+).” International Journal of Design & Nature and Ecodynamics 17, no. 3: 417–422. 10.18280/ijdne.170312.

[wer70385-bib-0025] Pandey, N. , J. Chandra , R. Xalxo , and K. Sahu . 2021. “Concept and Types of Phytoremediation.” In Approaches to the Remediation of Inorganic Pollutants, 281–302. Springer Singapore. 10.1007/978-981-15-6221-1_14.

[wer70385-bib-0026] Pandiarajan, S. , and V. Sankararajan . 2025. “A Review of Advanced Solutions in Constructed Wetlands for Sustainable Wastewater Management.” Physics of Fluids 37, no. 1: 011303. 10.1063/5.0244570.

[wer70385-bib-0041] Petrobras . 2025. “Relatório de Sustentabilidade 2024: Gestão de resíduos e descomissionamento.” Petrobras. https://sustentabilidade.petrobras.com.br/w/gestao‐de‐residuos‐e‐descomissionamento‐sustentavel.

[wer70385-bib-0027] Ram, B. K. , Y. Han , G. Yang , Q. Ling , and F. Dong . 2019. “Effect of Hexavalent Chromium [Cr (VI)] on Phytoremediation Potential and Biochemical Response of Hybrid Napier Grass With and Without EDTA Application.” Plants 8, no. 11: 515. 10.3390/plants8110515. 31744206 PMC6918263

[wer70385-bib-0028] Román‐Ramos, A. E. , C. E. Aucique‐Perez , D. Debona , and L. J. Dallagnol . 2024. “Nitrogen and Silicon Contribute to Wheat Defense's to *Pyrenophora tritici‐repentis*, but in an Independent Manner.” Plants 13, no. 11: 1426. 10.3390/plants13111426. 38891235 PMC11174962

[wer70385-bib-0029] Schröder, P. , L. Lyubenova , and C. Huber . 2009. “Do Heavy Metals and Metalloids Influence the Detoxification of Organic Xenobiotics in Plants?” Environmental Science and Pollution Research 16, no. 7: 795–804. 10.1007/s11356-009-0168-7. 19462193

[wer70385-bib-0030] Štolfa, I. , T. Ž. Pfeiffer , D. Špoljarić , T. Teklić , and Z. Lončarić . 2015. “Heavy Metal‐Induced Oxidative Stress in Plants: Response of the Antioxidative System.” In Reactive Oxygen Species and Oxidative Damage in Plants Under Stress, 127–163. Springer International Publishing. 10.1007/978-3-319-20421-5_6.

[wer70385-bib-0031] Swaefy, H. , R. El‐Ziat , and H. Khater . 2020. “The Ability of Red Fountain Grass to Grow in Cadmium‐Contaminated Soil.” Journal of Plant Production 11, no. 1: 17–23. 10.21608/jpp.2020.77986.

[wer70385-bib-0032] Tan, H. W. , Y. L. Pang , S. Lim , and W. C. Chong . 2023. “A State‐of‐the‐Art of Phytoremediation Approach for Sustainable Management of Heavy Metals Recovery.” Environmental Technology and Innovation 30: 103043. 10.1016/j.eti.2023.103043.

[wer70385-bib-0033] Tanwir, K. , Amna , M. T. Javed , M. Shahid , M. S. Akram , and Q. Ali . 2021. “Antioxidant Defense Systems in Bioremediation of Organic Pollutants.” In Handbook of Bioremediation, 505–521. Elsevier. 10.1016/B978-0-12-819382-2.00032-6.

[wer70385-bib-0034] Taufikurahman, T. , L. Melani , N. L. H. Sianturi , N. K. D. A. Wirawan , and A. F. Saifullah . 2023. “Effective Removal of Chromium From Contaminated Water Using Horizontal Subsurface Flow Constructed Wetland With Napier Grass (*Pennisetum purpureum*).” Current Research on Biosciences and Biotechnology 4, no. 2: 275–282. 10.5614/crbb.2023.4.2/226W5GYH.

[wer70385-bib-0035] Thakur, T. , M. Barya , J. Dutta , et al. 2023. “Integrated Phytobial Remediation of Dissolved Pollutants From Domestic Wastewater Through Constructed Wetlands: An Interactive Macrophyte‐Microbe‐Based Green and Low‐Cost Decontamination Technology With Prospective Resource Recovery.” Water 15, no. 22: 3877. 10.3390/w15223877.

[wer70385-bib-0036] Wagner, T. V. , F. Al‐Manji , J. Xue , et al. 2021. “Effects of Salinity on the Treatment of Synthetic Petroleum‐Industry Wastewater in Pilot Vertical Flow Constructed Wetlands Under Simulated Hot Arid Climatic Conditions.” Environmental Science and Pollution Research 28, no. 2: 2172–2181. 10.1007/s11356-020-10584-8. 32875449 PMC7785543

[wer70385-bib-0037] Wakeel, A. , M. Xu , and Y. Gan . 2020. “Chromium‐Induced Reactive Oxygen Species Accumulation by Altering the Enzymatic Antioxidant System and Associated Cytotoxic, Genotoxic, Ultrastructural, and Photosynthetic Changes in Plants.” International Journal of Molecular Sciences 21, no. 3: 728. 10.3390/ijms21030728. 31979101 PMC7037945

[wer70385-bib-0038] Wang, X. , H. Liu , F. Yu , et al. 2019. “Differential Activity of the Antioxidant Defence System and Alterations in the Accumulation of Osmolyte and Reactive Oxygen Species Under Drought Stress and Recovery in Rice (*Oryza sativa* L.) Tillering.” Scientific Reports 9, no. 1: 8543. 10.1038/s41598-019-44958-x. 31189967 PMC6561971

[wer70385-bib-0039] Wang, X. , B. Yao , and X. Su . 2018. “Linking Enzymatic Oxidative Degradation of Lignin to Organics Detoxification.” International Journal of Molecular Sciences 19, no. 11: 3373. 10.3390/ijms19113373. 30373305 PMC6274955

[wer70385-bib-0040] Watzinger, A. , M. Hager , T. Reichenauer , G. Soja , and P. Kinner . 2021. “Unravelling the Process of Petroleum Hydrocarbon Biodegradation in Different Filter Materials of Constructed Wetlands by Stable Isotope Fractionation and Labelling Studies.” Biodegradation 32, no. 3: 343–359. 10.1007/s10532-021-09942-1. 33860902 PMC8134294

